# PRMT5 promotes psoriatic inflammation through mediating dimethylation of p65 in keratinocytes

**DOI:** 10.1186/s10020-026-01558-6

**Published:** 2026-07-10

**Authors:** Xiaoru Hu, Yuzi Tian, Qingyu Hong, Wanyao Liu, Weina Li, Jia Guo

**Affiliations:** 1 Department of Rheumatology and Immunology, Xiangya Hospital, Central South University, Changsha, Hunan China; 2 Provincial Clinical Research Center for Rheumatic and Immunologic Diseases, Xiangya Hospital, Changsha, Hunan China; 3 National Clinical Research Center for Geriatric Disorders, Xiangya Hospital, Central South University, Changsha, Hunan China; 4 Department of Dermatology, Xiangya Hospital, Central South University, No.87 Xiangya Road, Changsha, 410008 Hunan China; 5 National Engineering Research Center of Personalized Diagnostic and Therapeutic Technology, Changsha, 410008 Hunan China; 6 Hunan Key Laboratory of Skin Cancer and Psoriasis, Changsha, 410008 Hunan China; 7 Hunan Engineering Research Center of Skin Health and Disease, Changsha, 410008 Hunan China

**Keywords:** PRMT5, Symmetric Dimethylation, p65, Psoriatic Inflammation, Keratinocyte, Pharmacological inhibition, EPZ015666

## Abstract

**Background:**

Psoriasis is a chronic inflammatory skin disease driven by aberrant keratinocyte activation and immune dysregulation. Protein arginine methyltransferase 5 (PRMT5) was identified as a candidate regulator in psoriatic keratinocytes, yet its function and mechanistic role in psoriasis pathogenesis remain unclear.

**Methods:**

We analyzed multiple public bulk RNA-sequencing and single-cell RNA-sequencing (scRNA-seq) transcriptomic datasets from psoriasis patients to assess PRMT5 mRNA expression and distribution. Protein expression was further validated by immunofluorescence staining of psoriatic lesion biopsies. Functional validation employed siRNA-mediated *Prmt5* knockdown in imiquimod-induced psoriasis model. Transcriptomic profiling combined with in vitro human primary keratinocytes experiments was performed with *PRMT5* knockdown or inhibition to map downstream potential mechanisms. Specific mechanistic investigations included co-immunoprecipitation, molecular docking, GST pull-down, mass spectrometry, and site-directed mutagenesis to characterize the PRMT5-p65 interaction and symmetric dimethylarginine (SDMA) modification. Finally, the therapeutic potential of PRMT5 inhibition by EPZ015666 was evaluated in imiquimod and IL-23-induced psoriasis models.

**Results:**

PRMT5 was markedly upregulated in psoriatic keratinocytes. Knockdown of *Prmt5* reduces local inflammation responses in the imiquimod-induced mouse model. Transcriptomic profiling combined with in vitro detection defined that inhibition of PRMT5 can dampen the inflammatory response of keratinocytes. Mechanistically, PRMT5 inhibition markedly reduces p65 protein levels, prompting further exploration of the PRMT5-p65 axis. Co-immunoprecipitation, molecular docking and GST pull down assay confirmed a direct PRMT5-p65 interaction; PRMT5 knockdown attenuated this interaction, diminishes symmetric dimethylarginine (SDMA) modification of p65, and compromises p65 protein stability. Accordingly, p65 overexpression restored inflammatory cytokine production in PRMT5-knockdown keratinocytes, functionally validating this regulatory axis. Mass spectrometry combined with site-directed mutation experiments revealed that PRMT5 facilitates p65 symmetric dimethylation at R158 and R166, which suppresses autophagy-mediated lysosomal degradation, thereby promoting p65-dependent cytokines expression. Pharmacological inhibition of PRMT5 with EPZ015666 ameliorates psoriatic inflammatory responses in vivo, underscoring its translational potential value.

**Conclusions:**

These findings establish a PRMT5-p65 regulatory axis wherein PRMT5-mediated symmetric dimethylation at R158 and R166 stabilizes p65 by autophagy-mediated lysosomal degradation and amplifies NF-κB-driven inflammation in keratinocytes, identifying PRMT5 as a potential therapeutic target in psoriasis.

**Graphical Abstract:**

**Figure:**
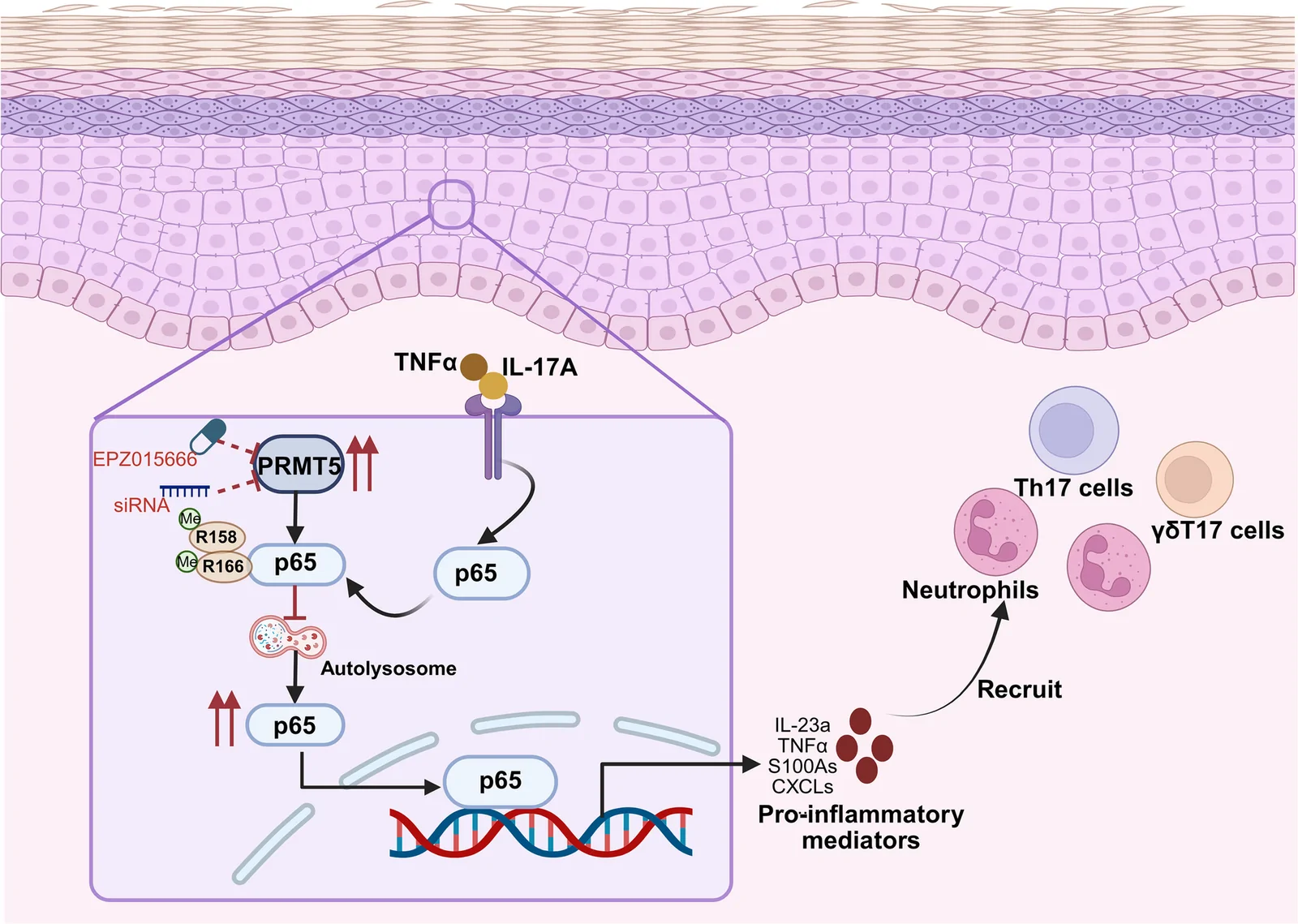

**Supplementary Information:**

The online version contains supplementary material available at https://doi.org/10.1186/s10020-026-01558-6.

## Background

Psoriasis is a chronic immune-mediated inflammatory disease affecting approximately 2–3% of the global population, characterized by aberrant keratinocyte hyperproliferation, immune cell infiltration, and sustained inflammatory responses (Armstrong and Read 2020; Armstrong 2025; Griffiths et al. 2021). The pathogenesis of psoriasis involves complex interactions between keratinocytes and the immune system (Hawkes et al. 2017; Lowes et al. 2013). Upon cytokine stimulation, keratinocytes produce a broad repertoire of chemokines, antimicrobial peptides, and pro-inflammatory mediators that orchestrate the recruitment and activation of infiltrating immune cells, thereby establishing and perpetuating the self-amplifying inflammatory circuit that defines psoriatic pathology (Zhou et al. 2022).

Central to this keratinocyte-driven inflammatory program is the transcription factor NF-κB-in particular its RelA (p65) subunit, which serves as the principal transducer of upstream cytokine signals into downstream gene expression. In psoriatic keratinocytes, canonical NF-κB signaling is robustly activated by inflammatory stimulation, driving transcription of key pro-inflammatory targets including IL-6, IL-8, CXCL1, and CCL20, thereby sustaining immune cell infiltration and epidermal remodeling (Liu 2024; Goldminz et al. 2013). The transcriptional activity of p65 is intricately regulated at the post-translational level through phosphorylation, acetylation, ubiquitination, and sumoylation, each governing discrete aspects of its stability, nuclear translocation, promoter binding, and co-activator recruitment (Cildir et al. 2016; Perkins 2006). Arginine methylation has recently emerged as a pivotal post-translational modification that governs a broad range of biological processes, including protein stability, RNA processing, transcriptional regulation, subcellular localization, DNA damage response, and cell cycle progression (Blanc and Richard 2017; Guccione and Richard 2019). Nevertheless, whether arginine methylation functionally regulates p65 activity within the context of keratinocyte-driven inflammation in psoriasis has not been previously investigated.

Protein arginine methyltransferases (PRMTs) comprise a family of nine mammalian members classified into three types according to their catalytic products: Type I PRMTs (PRMT1, 3, 4, 6, and 8) generate asymmetric dimethylarginine (ADMA), Type II PRMTs (PRMT5 and 9) produce symmetric dimethylarginine (SDMA), and the sole Type III member PRMT7 catalyzes monomethylation (Blanc and Richard 2017). Among these, PRMT5 is the predominant type II arginine methyltransferase responsible for catalyzing the symmetric dimethylation of arginine residues (Rme2s) on both histone and non-histone substrates (Stopa et al. 2015). On chromatin, PRMT5-mediated symmetric dimethylation of histone H4 at arginine 3 (H4R3me2s) is classically associated with transcriptional repression; however, accumulating evidence has demonstrated that PRMT5 exerts pleiotropic effects through the modification of non-histone substrates involved in RNA splicing, DNA damage repair, cell cycle progression, and cytokine signaling (Zhao 2009; Yang and Bedford 2013). PRMT5 is aberrantly overexpressed across a spectrum of malignancies and chronic inflammatory disorders, where it facilitates oncogenic transcription and immune evasion through context-dependent substrate methylation (Fong 2019; Kryukov 2016). Emerging evidence suggests that PRMT5 can affect transcriptional factors, including NF-κB, STAT3, or c-myc signaling cascades, modulating their activity through direct protein methylation or by remodeling chromatin accessibility at inflammatory gene loci (Abe et al. 2024; Cai 2021; Liu 2020; Wei 2013). These observations raise the compelling but previously untested possibility that PRMT5-mediated arginine methylation may modulate p65 activity to amplify NF-κB-dependent inflammation in psoriatic keratinocytes.

In the present study, we integrated bulk RNA sequencing, single-cell transcriptomic profiling across multiple independent public cohorts, and immunofluorescence analysis of human psoriatic skin biopsies to demonstrate that PRMT5 is significantly upregulated in psoriatic keratinocytes. Through complementary pharmacological inhibition with EPZ015666 and siRNA-mediated silencing in both murine psoriasis models and primary human keratinocytes, we establish that PRMT5 is functionally required for the maintenance of epidermal inflammatory responses, neutrophil and Th17 cell recruitment, and pro-inflammatory cytokine production. Mechanistically, transcriptomic profiling of PRMT5-inhibited keratinocytes implicated inflammatory signaling as the principal downstream effector pathway. Co-immunoprecipitation and molecular docking analyses confirmed a direct physical interaction between PRMT5 and p65; PRMT5 loss reduced p65 protein abundance and attenuated transcriptional activity of p65-dependent genes. Critically, mass spectrometry identified a previously unreported PRMT5-mediated symmetric dimethylation of p65 at arginine residues R158 and R166. Site-directed mutagenesis of these residues rendered p65 refractory to PRMT5-mediated enhancement of cytokine expression, and rescue experiments confirmed that p65 re-expression restores the inflammatory responses suppressed by PRMT5 silencing. Together, these findings delineate a PRMT5-p65 R158/R166me2s regulatory axis as a critical post-translational mechanism amplifying NF-κB-dependent inflammatory transcription in psoriatic keratinocytes, and identify PRMT5 as a mechanistically grounded and therapeutically actionable target for psoriasis intervention.

## Method and materials

### Public dataset retrieval and bioinformatic analysis

Gene expression microarray and RNA sequencing (RNA-seq) datasets related to psoriasis, together with corresponding clinical metadata, were obtained from the Gene Expression Omnibus (GEO) repository. For lesional versus non-lesional (LL vs. NL) comparisons, fourteen datasets were retrieved (GSE106992, GSE117239, GSE136757, GSE26866, GSE30999, GSE34248, GSE41662, GSE41663, GSE51440, GSE6710, GSE67785, GSE67853, GSE69967, GSE85034). Additional four datasets (GSE14905, GSE52471, GSE53431, GSE54456) were used for psoriatic lesional (PL) versus healthy normal skin comparisons. All downstream bioinformatic analyses were conducted in R (v4.2.1) using relevant Bioconductor packages.

### Single-Cell RNA Sequencing (ScRNA-seq) analysis

ScRNA-seq data from GEO (GSE173706), encompassing skin biopsies from healthy controls (HC), psoriasis non-lesional (PN), and psoriasis lesional (PL) samples, were processed using the Seurat package (v4.0) in R. Quality control retained only cells with 200–5,000 detected genes and mitochondrial gene fractions below 15%. Dimensionality reduction was performed via UMAP, and unsupervised graph-based clustering was applied to define cell populations, which were subsequently annotated based on canonical marker gene expression. PRMT5 expression was visualized across sample groups using UMAP feature plots. Within the keratinocyte compartment, PRMT5 expression levels were compared among HC, PN, and PL groups using violin plots, with statistical significance assessed by the Wilcoxon rank-sum test.

### Multi-color immunohistochemistry assay

Formalin-fixed, paraffin-embedded tissue Sects. (5 μm) were deparaffinized in xylene and rehydrated through a graded ethanol series. Antigen retrieval was carried out in citrate buffer (pH 6.0) by microwave irradiation (100–150 mW, 20 min). Non-specific binding was blocked with 5% goat serum (ZLI-9022; ZSGB-Bio, China) for 30 min at room temperature (RT). Sections were co-incubated overnight at 4 °C with primary antibodies against PRMT5 (1:400; 79998S; Cell Signaling Technology) and Pan-Cytokeratin (1:400; 4545; Cell Signaling Technology). Following washing, a secondary antibody (PV-9000; ZSGB-Bio, China) was applied for 30 min at RT, and signal was amplified sequentially via tyramide signal amplification (TSA) using TYR-570 nm (for PRMT5) and TYR-690 nm (for Pan-CK) fluorescent reagents (AFIHC027; AiFang Biological), each incubated for 10 min at RT in the dark. The same staining protocol was applied for Ly6g (1:200; ab238132; Abcam) and CD4 (1:200; 48,274; Cell Signaling Technology), using TYR-570 nm (for Ly6g) and TYR-690 nm (for CD4) fluorescent reagents. For multiplexed imaging, a second round of antigen retrieval (citrate buffer, pH 6.0; 100–150 mW, 15 min) was performed to strip antibodies before each subsequent staining cycle. Nuclear counterstaining with DAPI (ab104139; Abcam) was performed for 10 min at RT. Stained slides were digitally acquired using the Vectra® Polaris™ Automated Quantitative Pathology Imaging System (PerkinElmer) in fluorescence mode.

### LC–MS/MS-based mapping of symmetric dimethylarginine sites

#### Sample preparation

To identify PRMT5-dependent symmetric dimethylarginine (SDMA) sites on substrate proteins under inflammatory conditions, primary keratinocytes with or without PRMT5 knockdown and stimulated with TNF-α and IL-17A were subjected to co-immunoprecipitation (co-IP) using an anti-PRMT5 antibody (79,998; Cell Signaling Technology). Co-IP was used to enrich PRMT5-interacting proteins prior to mass spectrometry, enabling targeted identification of SDMA modification sites within the PRMT5 substrate complex. Immunoprecipitates were reduced with TCEP, alkylated with chloroacetamide, and precipitated with methanol using HIPure Viral Mini Column (M024-00, Magen) with a glass fiber filter membrane (pore size: 0.7 μm). Protein pellets were resuspended in TEAB buffer and digested overnight with trypsin at 37 °C. Resulting peptides were desalted using C18 solid-phase extraction cartridges (Qinglian Bio, Beijing, China). The cartridges were activated with acetonitrile, equilibrated with 0.1% formic acid, and loaded with the peptide samples. After washing with 0.1% formic acid to remove impurities, peptides were eluted with 40% acetonitrile and dried under vacuum.

#### LC–MS/MS analysis

Nanoflow LC–MS/MS analysis of tryptic peptides was conducted on a quadrupole Orbitrap mass spectrometer (Q Exactive HF-X, Thermo Fisher Scientific, USA) coupled to an EASY nLC 1200 ultra-high pressure system (Thermo Fisher Scientific) via a nano-electrospray ion source. 1 μg of peptides was loaded on a 25 cm column (100 μm inner diameter, packed using ReproSil-Pur C18-AQ 1.5- µm silica beads; QL-HPLC-100*15; Beijing Qinglian Biotech Co., Ltd, Beijing, China). Peptides were separated using a gradient from 8 to 12% B in 2 min, then 12% to 30% B in 3 min, and stepped up to 40% in 7 min, followed by a 10 min wash at 95% B at 300 nl per minute where solvent A was 0.1% formic acid in water and solvent B was 80% ACN and 0.1% formic acid in water. The total duration of the run was 30 min. Column temperature was kept at 60 °C using an in-house-developed oven. Briefly, the mass spectrometer was operated in “top-40” data-dependent mode, collecting MS spectra in the Orbitrap mass analyzer (120,000 resolution, 350–1500 m/z range) with an automatic gain control (AGC) target of 3E6 and a maximum ion injection time of 80 ms. The most intense ions from the full scan were isolated with an isolation width of 1.6 m/z. Following higher-energy collisional dissociation (HCD) with a normalized collision energy (NCE) of 27, MS/MS spectra were collected in the Orbitrap (15,000 resolution) with an AGC target of 5E4 and a maximum ion injection time of 45 ms. Precursor dynamic exclusion was enabled with a duration of 16 s.

#### Protein identification and quantitation

Raw files were analyzed using Proteome Discoverer (Thermo Fisher Scientific) with MS2 spectra searched against the UniProtKB human Swiss-Prot database using the Sequest HT engine. Search parameters included fully tryptic specificity, up to two missed cleavages, fixed carbamidomethylation of cysteine (+ 57.021 Da), variable oxidation of methionine (+ 15.995 Da), and precursor/fragment mass tolerances of 15 ppm and 0.02 Da, respectively. Peptide and protein false discovery rates were controlled at 1% using Percolator. Unique and razor peptides were used for quantification.

### Structural prediction and molecular docking

To predict the structural basis of the PRMT5–p65 interaction, the three-dimensional structure of human p65 was retrieved from the AlphaFold Protein Structure Database, and the crystal structure of human PRMT5 was obtained from the RCSB Protein Data Bank (PDB ID: 5C9Z) (Singh et al. 2024; Fleming 2025). Rigid-body protein–protein docking was performed using the GRAMM web server at physiological pH (7.4). The top 10 docking conformations (binding energies: − 624 to − 593 kcal/mol) were retained for analysis, and the top-ranked pose (binding energy: − 624 kcal/mol) was selected for downstream characterization. Protein–protein interaction interfaces were subsequently characterized using PLIP (Protein–Ligand Interaction Profiler), which identified hydrogen bonds, hydrophobic contacts, and salt bridges formed between p65 and PRMT5 across the candidate complexes (Schake et al. 2025). Three-dimensional structural representations of the docking complexes were generated and visualized using PyMOL v2.6 (Schrödinger, New York, NY, USA) (Lill and Danielson 2011).

### RNA Sequencing (RNA-seq) and transcriptomic analysis

To investigate the transcriptional landscape modulated by PRMT5 inhibition under inflammatory conditions, primary human keratinocytes stimulated with TNF-α (10 ng/ml) and IL-17A (20 ng/ml) were treated with or without EPZ015666 and subjected to bulk RNA sequencing with three biological replicates per group. Total RNA was extracted using TRIzol reagent (Invitrogen), and RNA integrity was assessed on an Agilent 2100 Bioanalyzer; only samples with an RNA Integrity Number (RIN) ≥ 8.0 were included. Sequencing libraries were prepared from poly-A-enriched mRNA using a strand-specific library preparation protocol, with size selection for fragments of 370–420 bp using AMPure XP beads (Beckman Coulter), followed by PCR amplification and sequencing on the Illumina NovaSeq 6000 platform. Clean data output ranged from 6.40 to 8.21 GB per sample across all six libraries (TNF-α + IL-17A group: 6.65, 6.93, and 8.21 GB; TNF-α + IL-17A + EPZ015666 group: 6.40, 7.17, and 6.93 GB). Read counts mapped to each gene were quantified from raw BAM files using FeatureCounts (v2.0.6), based on the alignment of clean reads to the human reference genome (GRCh38/hg38). FPKM values were then calculated for each gene based on gene length and total mapped read depth. Log2-transformed FPKM values were then used as input for differential expression analysis, which was performed using the R package limma (v3.64.3). All samples were prepared and sequenced within a single experimental batch; therefore, batch correction was not performed. Genes meeting thresholds of adjusted p < 0.05 and |log₂ (fold change) |> 1 were defined as differentially expressed genes (DEGs). Functional enrichment of DEGs was performed using the clusterProfiler package, encompassing Gene Ontology (GO) term enrichment and Kyoto Encyclopedia of Genes and Genomes (KEGG) pathway analysis. An adjusted p-value < 0.05 was applied as the significance threshold for all enrichment analyses.

### Cell lines and primary cell culture

The HaCaT immortalized human keratinocytes (provided by Caroline Le Poole, Loyola University Chicago, IL, USA) were maintained in RPMI-1640 medium (VivaCell, Shanghai, China) supplemented with 10% fetal bovine serum (12J262, ExCell Bio, China) and 1% penicillin–streptomycin mix at 37℃ in the presence of 5% CO2.

Primary human keratinocytes were isolated from foreskin tissue obtained from healthy donors undergoing elective circumcision, with written informed consent secured from all donors before tissue collection. Briefly, foreskin specimens were incubated overnight at 4 °C in dispase II solution (2 mg/ml; D4693, Sigma-Aldrich) to facilitate epidermal-dermal separation. Following mechanical peeling, the epidermal sheet was rinsed with ice-cold DPBS and subjected to trypsin digestion at 37 °C for 4 min. The reaction was quenched with DMEM containing 10% FBS, and the resulting cell suspension was washed twice with DPBS. Pelleted cells were resuspended in KGM Keratinocyte Growth Medium BulletKit (192,060; Lonza) and seeded onto collagen I-coated plates (cat. no. A10483-01; Gibco) for culture under identical conditions (37 °C, 5% CO₂).

### Pharmacological Inhibition and cell treatment

Primary keratinocytes (3.5 × 10^5^ primary/well) were seeded into a 12-well plate. For PRMT5 inhibition, cells were treated with EPZ015666 (HY-N0516, MedChemExpress) at 5 μM for 24 h. For protein degradation analysis, cells were first transfected with si-PRMT5. At 48 h post-transfection, the culture medium was replaced with fresh medium containing 100 μg/mL cycloheximide (CHX, S7418, Selleck). Cells were then harvested at 0, 4, 8, and 12 h after CHX addition. In separate experiments, at 48 h after si‑PRMT5 transfection, cells were treated with either 10 μM MG132 (S2619, Selleck) or 50 μM hydroxychloroquine (CQ, E4824, Selleck). Cells were collected at 6 h following inhibitor treatment. Cells were subsequently harvested for quantitative RT-PCR or western blotting analyses.

### Cell transfections and rescue assay

For gene silencing, primary keratinocytes were transfected with siRNA using Lipofectamine™ RNAiMAX (13,778,150; ThermoFisher Scientific) in Keratinocyte Growth Medium for 48 h following the manufacturer's protocol. For plasmid overexpression, plasmid transfection was performed using TurboFect™ Transfection Reagent (R0533, ThermoFisher Scientific) under identical conditions. Transfection efficiency was assessed at both the protein and mRNA levels.

For the rescue assay, primary keratinocytes were first transfected with si-*PRMT5.*

(Sense:5’-CAGAGAUCCUAUGAUUGACAA-3’, Antisense5’- UUGUCAAUCAUAGGAUCUCUG −3’) or si-*NC* for 48 h, followed by p65 plasmid transfection for an additional 24 h, after which cells were collected for quantitative RT-PCR.

### Plasmid constructs

Human PRMT5 cDNA (NM_006109) and human RELA cDNA (NM_021975) were chemically synthesized and verified by DNA sequencing at Changsha Youze Biotechnology Co., Ltd. The PRMT5 fragment was then subcloned into the pcDNA3.1-HA-C vector, and the wild‑type RELA fragment was subcloned into the pcDNA3.1-3xFlag vector, using standard restriction enzyme-based cloning methods. Three RELA point mutants (R336A, R158A, R166A) were generated via the QuikChange site‑directed mutagenesis kit (Agilent Technologies) using the wild‑type RELA plasmid as a template. All mutant constructs were confirmed by DNA sequencing to ensure the desired mutations and the absence of unwanted changes.

### Plasmid amplification and extraction

Plasmid constructs were transformed into E. coli DH5α cells by heat shock, and selected on ampicillin-containing (100 μg/ml) LB agar. Plasmid DNA was extracted from overnight cultures using the HiPure Plasmid Mini Kit (Magen) per the manufacturer's instructions. DNA concentration and purity were assessed by NanoDrop spectrophotometry; an A260/A280 ratio of 1.8–2.0 was required for downstream use.

### Quantitative RT-PCR

Total RNA was extracted from cells or tissue samples using MagZol reagent (R4801-01; Magen), and RNA concentration and purity were assessed using a NanoDrop instrument (ThermoFisher Scientific). Equal amounts of RNA were reverse-transcribed using HiScript Q RT (K1622; ThermoFisher Scientific) in accordance with the manufacturer's instructions. Quantitative PCR was performed with SYBR Green PCR Master Mix (B21703; Bimake) on a real-time PCR system, with β-actin as the internal reference. Relative mRNA expression was calculated using the 2⁻ΔΔCt method. The sequences of primers used in this study were as follows:

**Table:** 

Primer	Forward (5’−3’)	Reverse (5’−3’)
*β-Actin* (h)	ACTGGAACGGTGAAGGTGAC	AGAGAAGTGGGGTGGCTTTT
*IL-23A* (h)	CTGAGGGTCACCACTGGGAGAC	TGGAGGCTGCGAAGGATTTTGAAG
*TNF-α*(h)	CTCTTCTGCCTGCTGCACTTTG	ATGGGCTACAGGCTTGTCACTC
*S100A7* (h)	CCCAACTTCCTTAGTGCCTGTGAC	GCTCTGCTTGTGGTAGTCTGTGG
*S100A8* (h)	TTGCTAGAGACCGAGTGTCCTCAG	GCCACGCCCATCTTTATCACCAG
*S100A9* (h)	GAACACATCATGGAGGACCTGGAC	GGTTAGCCTCGCCATCAGCATG
*CXCL1*(h)	CTGCTGCTCCTGCTCCTGGTAG	CGTTCACACTTTGGATGTTCTTGGG
*CXCL2*(h)	CCCAAACCGAAGTCATAGCCACAC	GATTTTCTTAACCATGGGCGATGCG
*CXCL3*(h)	AACCGAAGTCATAGCCACACTCAAG	CAGTTGGTGCTCCCCTTGTTCAG
*RELA*(h)	TGAACCGAAACTCTGGCAGCTG	CATCAGCTTGCGAAAAGGAGCC
*PRMT5*(h)	GCACCAACTACACACACAGA	AGCCACTGCAATCCTCTTAC
*Prmt5*(m)	CCTGCTTTACCTTCAGCCATCC	GCACAGTCTCAAAGTAGCCTGC
*β-actin* (m)	CATTGCTGACAGGATGCAGAAGG	TGCTGGAAGGTGGACAGTGAGG
*S100a8* (m)	CAAGGAAATCACCATGCCCTCTA	ACCATCGCAAGGAACTCCTCGA
*S100a9* (m)	TGGTGGAAGCACAGTTGGCAAC	CAGCATCATACACTCCTCAAAGC
*Il-23* (m)	GGACTCAAGGACAACAGCCAGTTC	TGAAGATGTCAGAGTCAAGCAGGTG
*Il-17a* (m)	CAGACTACCTCAACCGTTCCAC	TCCAGCTTTCCCTCCGCATTGA
*Tnf-α* (m)	CGCTCTTCTGTCTACTGAACTTCGG	GTGGTTTGTGAGTGTGAGGGTCTG
*Cxcl1* (m)	CTGCTGCTCCTGCTCCTGGTAG	CGTTCACACTTTGGATGTTCTTGGG
*Cxcl3* (m)	GCCACTCTCAAGGATGGTCAAGAAG	GGACTTGCCGCTCTTCAGTATCTTC

### Western blotting

Cells were lysed in RIPA buffer (P0013C; Beyotime) supplemented with protease inhibitor (B14002, Bimake) and phosphatase inhibitor (B15002, Bimake). Protein concentrations were determined, and 10–30 μg of total protein per lane was resolved on 10–12% SDS-PAGE gels, then transferred onto PVDF membranes. After blocking with 5% BSA or 5% milk in TBST, the membranes were incubated overnight at 4 °C with primary antibodies against GAPDH (60,004-I-Ig, Proteintech Group); PRMT5 (79998 T; Cell Signaling Technology); p65 (T55034; Abmart); FLAG (66,008–4-Ig, Proteintech Group); GST (10,000–0-AP, Proteintech Group); His (66,005–1-Ig, Proteintech Group) and Symmetric Di-Methyl Arginine Motif [sdme-RG] (SDMA, 13222 T, Cell Signaling Technology). Following TBST washes, membranes were incubated with goat anti-mouse (AS003; ABclonal) or goat anti-rabbit (AS014; ABclonal) secondary antibodies. Signals were detected using an NcmECL Ultra kit (P10300; NCM Biotech) and imaged on an Odyssey® Fc Imaging System (LI-COR Biosciences, Lincoln, NE, USA).

### Psoriasis-like mouse model

Eight-week-old female BALB/c mice were purchased from SLAC Laboratory Animal Co., Ltd. (Changsha, China) and housed under specific-pathogen-free conditions with a 12 h light/dark cycle and ad libitum access to food and water. All procedures were approved by the Institutional Animal Care and Use Committee of Xiangya Hospital, Central South University.

For IMQ-induced psoriasis-like skin inflammation model, 62.5 mg imiquimod (IMQ) cream (120,503; Sichuan Mingxin Pharmaceutical) was applied topically daily to shaved dorsal skin for five consecutive days. Disease severity was monitored daily using a modified PASI scoring system evaluating erythema, scaling, and skin thickness, each scored on a 0–4 scale (0 = none; 4 = severe). PASI scoring and histological assessment were performed by investigators who were blinded to group allocation. For pharmacological inhibition, EPZ015666 (50 mg/kg) in corn oil was gavaged once daily concurrent with IMQ application. For RNA interference-based knockdown, drug preparation of 2.5 nmol siRNA and Lipofectamine 3000 in 20 mg of water-based gel for topical application of either *Prmt5* siRNA (Sequence: 5′GCACAGTTTGAGATGCCTTAT3′) or negative control siRNA was applied to either side of shaved back skin (1.5 cm × 2 cm area) two days prior, on the day of, and two days after the start of IMQ treatment.

For the IL-23-induced psoriasis-like skin inflammation model, 25 μL PBS containing 500 ng recombinant mouse IL-23 (HY-P70709, MedChemExpress) was injected intradermally into the right ear every other day for 16 days. Disease severity was monitored daily by evaluating erythema. For pharmacological inhibition, EPZ015666 (50 mg/kg) dissolved in corn oil was administered by oral gavage on the same days as IL-23 injection.

### GST pull-down assay

To confirm that the interaction between PRMT5 and p65 is direct rather than mediated by a bridging protein, GST pull-down assay was performed using the GSTSep Glutathione MagBeads GST-tagged Protein Purification Kit (20562ES03,Yeason) according to the manufacturer's instructions. Recombinant GST-tagged human RELA/NF-κB p65 protein (12,054-H09E, aa 1–306, GST Tag; Sino Biological,) or recombinant Schistosoma japonicum GST protein (11,213-HNAE, Sino Biological) was used as bait protein, and recombinant human PRMT5 protein (11,074-H32B,His & FLAG Tag; Sino Biological) was used as prey protein. Bait proteins were bound to pre-equilibrated beads at room temperature for 30 min, washed, and then incubated with recombinant PRMT5 protein for an additional 30 min. After washing, bound proteins were eluted and analyzed by SDS-PAGE and immunoblots detecting by anti-His antibody and anti-GST antibody.

### Flow cytometry

Single-cell suspensions were isolated by enzymatic digestion of dorsal skin in a buffer containing Dispase (2 mg/ml), Collagenase IV (3 mg/ml), and DNase I at 37 °C for 2 h with continuous agitation at 200 rpm. Single-cell suspensions were obtained by passage through 40 μm strainers (352,340; BD Falcon) and washed twice with PBS. For intracellular cytokine detection, cells were stimulated with a cell stimulation cocktail containing protein transport inhibitors (00–4975-03; eBioscience) for 5 h at 37 °C. Following stimulation, live/dead discrimination was assessed using the Zombie Aqua™ Fixable Viability Kit (423,102; BioLegend; 1:500). Surface staining was performed with the following antibodies: APC/Cy7 anti-CD45 (103,116; BioLegend; 1:100), APC anti-CD3 (100,236; BioLegend; 1:100), BB700 anti-CD4 (566,407; BD Biosciences; 1:100), BV421 anti-CD11b (101,235; BioLegend; 1:100), Brilliant Violet 605™ anti-mouse TCR γ/δ (118,129, Biolegend; 1:100) and BV711 anti-Ly-6G (563,979; BD Biosciences; 1:100). Intracellular staining for IL-17A was conducted following fixation and permeabilization with the Foxp3/Transcription Factor Staining Buffer Set (00–5523-00; eBioscience) for 30 min at 4 °C, using PE anti-IL-17A (506,904; BioLegend; 1:100). Data were acquired on a BD LSRFortessa X-20 cytometer and analyzed with FlowJo v10.4.

### Statistical analyses

Statistical analyses were conducted using GraphPad Prism (version 10.6.0). For in vivo and in vitro studies, when the sample size was too small for normality testing, one-way ANOVA (one variable and three or more groups) and two-way ANOVA (two variable and three or more groups) with Tukey's multiple comparisons test were used to compare multiple groups, and an unpaired two-sided t-test was used to compare two groups. For experiments with larger sample sizes, normality was assessed using the Shapiro–Wilk test. When normality was confirmed, variance homogeneity was evaluated using Bartlett's test, followed by one-way ANOVA and two-way ANOVA with Tukey's multiple comparisons test. When normality was not met, the Wilcoxon rank-sum test was applied. A p-value less than 0.05 was considered significant for all experiments.

## Result

### PRMTT5 is upregulated in keratinocytes of psoriasis lesions and contributes to the psoriasis pathogenesis in a psoriatic mouse model

**Fig. 1:**
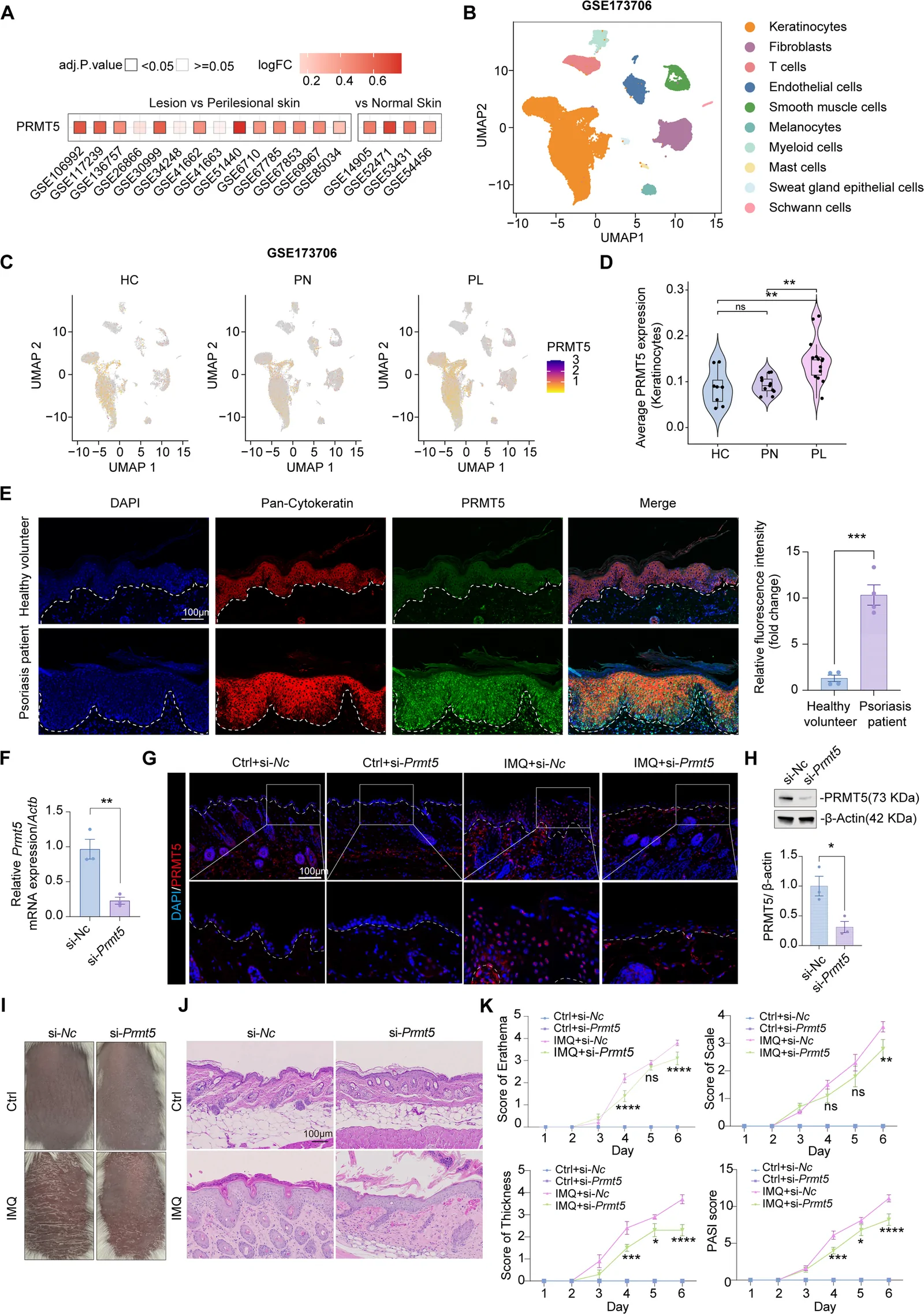
PRMT5 is upregulated in psoriatic keratinocytes and its knockdown alleviates psoriasis-mouse model phenotype. **A.** Heatmap showing PRMT5 expression (logFC) across multiple GEO datasets comparing lesional versus perilesional and normal skin. Red intensity reflects logFC magnitude; white indicates adj. P. **B.** UMAP visualization of single-cell RNA sequencing data (GSE173706) showing skin cell populations. UMAP, uniform manifold approximation and projection. **C** UMAP feature plots of PRMT5 expression in healthy controls (HC), psoriasis non-lesional (PN), and lesional (PL) skin. **D.** Violin plots comparing PRMT5 expression in keratinocytes across HC, PN, and PL groups. KC, keratinocytes. **E.** Immunofluorescence staining for DAPI, Pan-Cytokeratin, and PRMT5 in skin sections from healthy volunteers and psoriasis patients (scale bar = 100 μm), with quantification of relative PRMT5 fluorescence intensity (right). **F.** qRT-PCR validation of *Prmt5* expression in mouse primary keratinocytes transfected with Si-*Prmt5* or Si-*Nc*. **G** Immunofluorescence staining for PRMT5 in mouse dorsal skin treated with IMQ, combined with si-*Nc* or si-*Prmt5*. **H.** Reprensentative immunoblot of PRMT5 (upper) and quantification (lower) in mouse primary keratinocytes transfected with Si-*Prmt5* or Si-*Nc*. **I** Gross morphology of mouse dorsal skin treated with IMQ, combined with si-*Nc* or si-*Prmt5*. **J**.H&E staining of skin sections from the four treatment groups illustrating epidermal changes (scale bar = 100 μm). **K.** Time-course clinical scores (erythema, scale, thickness, and PASI) over 6 days across treatment groups. Data are presented as mean ± SEM (N = 3–4 for E, F, H and K). Statistical significance was determined by limma (A) and Wilcoxon rank-sum test (D), two-tailed unpaired Student’s t test (E, F, H) and two-way ANOVA followed by Tukey's multiple comparisons test (K). ns, not statistically significant. *p < 0.05, **p < 0.01, ***p < 0.001, ****p < 0.0001.

To investigate the role of PRMT5 in psoriasis, we first assessed its mRNA expression using publicly available bulk RNA-seq datasets. The results revealed that PRMT5 was significantly upregulated in lesional skin compared to normal or perilesional skin across almost all datasets (Fig. 1A). To obtain a high-resolution, unbiased perspective of its distribution, we subsequently interrogated public psoriatic single-cell RNA-sequencing (scRNA-seq) data. This analysis identified ten distinct cell populations, including keratinocytes, fibroblasts, T cells, endothelial cells, smooth muscle cells, melanocytes, myeloid cells, mast cells, sweat gland epithelial cells, and Schwann cells (Fig. 1B and Fig. S1A). Notably, PRMT5 expression was found to be significantly enriched specifically within the keratinocytes of psoriatic patients compared to normal or perilesional tissues (Figs. 1C, D and Fig. S1B). Consistent with these transcriptomic findings, immunofluorescence staining confirmed a substantial increase in PRMT5 protein levels in lesional skin, with the signal being most prominent in pan-cytokeratin (Pan-CK)-positive keratinocytes (Fig. 1E). Taken together, these findings demonstrate that PRMT5 is consistently and specifically upregulated in the keratinocytes of psoriatic lesions, suggesting its potential involvement in the pathogenic mechanisms of psoriasis.

To further elucidate the functional role of PRMT5 in driving psoriatic progression, we employed small interfering RNA (siRNA) to silence *Prmt5* expression in the dorsal skin of mice and then established an IMQ-induced skin inflammation mouse model. Transfection with si*-Prmt5* achieved efficient knockdown at both the mRNA and protein levels (Fig. 1F-H). We observed that knockdown of *Prmt5* significantly ameliorated IMQ-induced skin scaling, erythema, and thickening, resulting in a notable decrease of PASI score (Fig. 1I and Fig. 1K). Histopathological assessment confirmed that *Prmt5* knockdown led to a substantial improvement in epidermal thickening following the IMQ challenge (Fig. 1J). These data collectively indicate that PRMT5 is a critical mediator of psoriatic pathogenesis.

### Silencing *Prmt5* attenuates IMQ-induced skin inflammation phenotype in mouse model

**Fig. 2:**
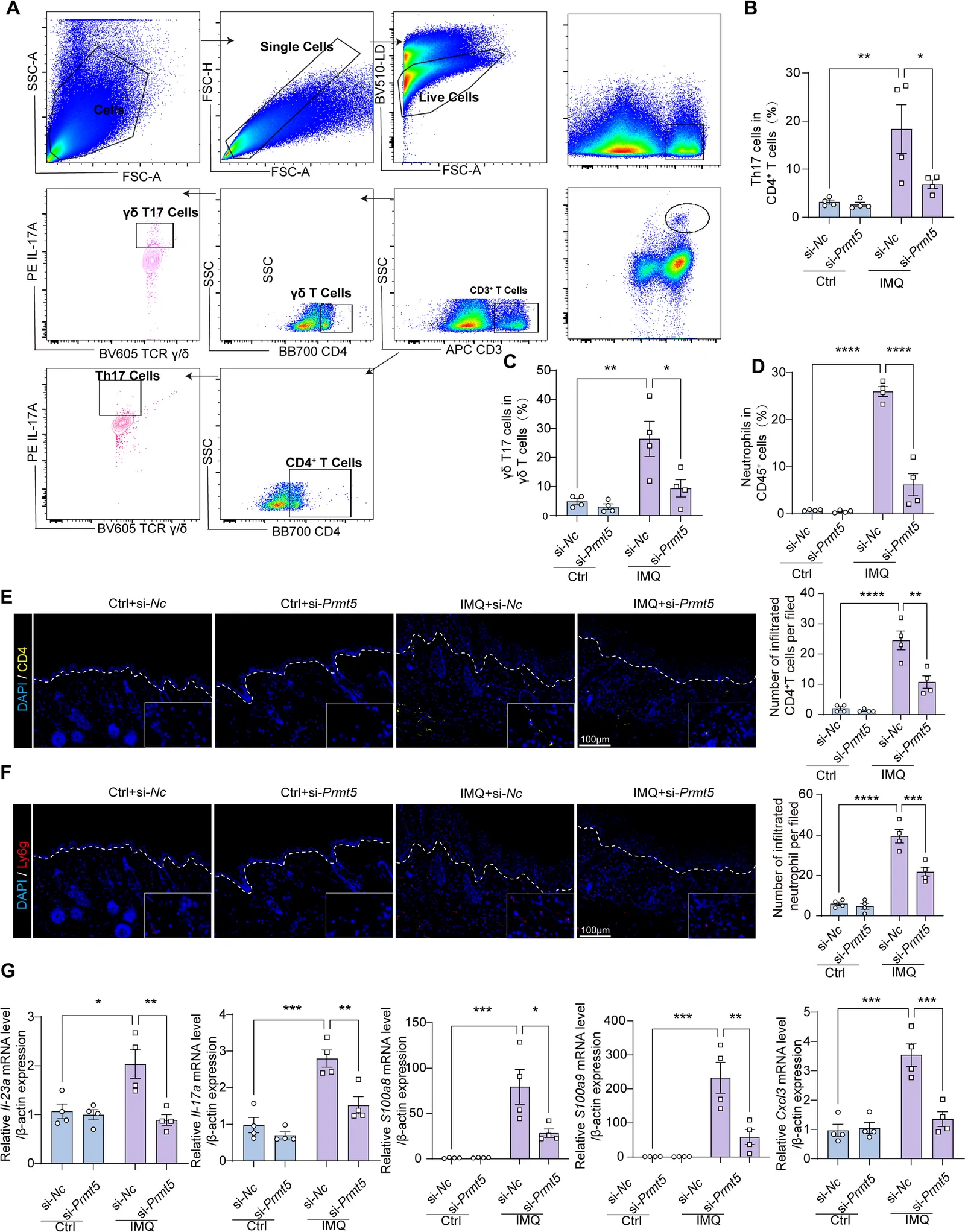
Knockdown of *Prmt5* inhibits the IMQ-induced inflammation responses **A** Gating strategy for flow cytometric analysis of immune cells in skin lesions. **B** Percentage of Th17 cells among infiltrating immune cells in lesional skin, assessed by flow cytometry. **C** Percentage of γδT17 cells among infiltrating immune cells in lesional skin, assessed by flow cytometry. **D** Percentage of neutrophils among infiltrating immune cells in lesional skin, assessed by flow cytometry. **E** Immunofluorescence staining for CD4 in mouse dorsal skin treated with IMQ, combined with si-*Nc* or si-*Prmt5*, with quantification of CD4^+^T cells number (right). **F** Immunofluorescence staining for Ly6G in mouse dorsal skin treated with IMQ, combined with si-*Nc* or si-*Prmt5*, with quantification of Neutrophils number (right). **G**. qRT-PCR analysis of inflammation-related genes mRNA expression in the affected dorsal skin. Data are presented as mean ± SEM (*N* = 4 for B-G). Statistical significance was determined by one-way ANOVA followed by Tukey's multiple comparisons test (B-G).ns, not statistically significant. *p < 0.05, **p < 0.01, ***p < 0.001, ****p < 0.0001

Psoriasis is fundamentally driven by inflammatory dysregulation, where multiple self-amplifying feedback loops within the cytokine network continuously fuel and accelerate the pathogenic circuits (Armstrong and Read 2020). We next assessed local immune cell infiltration by flow cytometry. The proportions of key pathogenic populations-neutrophils and IL-17A–producing T cells, such as the Th17 and γδT17 subset -were significantly reduced following si*-Prmt5* treatment (Fig. 2A–D). Additionally, immunofluorescence staining further confirmed reductions in CD4^+^ T cells and neutrophil infiltration (Fig. 2E and F). Consistent with these cellular changes, the expression of established psoriatic inflammatory factors, including *Il-17a, Il23a, Cxcl3, S100a8, and S100a9, was* significantly downregulated in si*-Prmt5*-treated lesional skin relative to the IMQ group (Fig. 2G). Collectively, these findings demonstrate that PRMT5 is functionally required for the maintenance of psoriatic inflammatory responses in *vivo*.

### Transcriptomic profiling identifies inflammatory signaling pathways as downstream targets of PRMT5 inhibition in keratinocytes

**Fig. 3:**
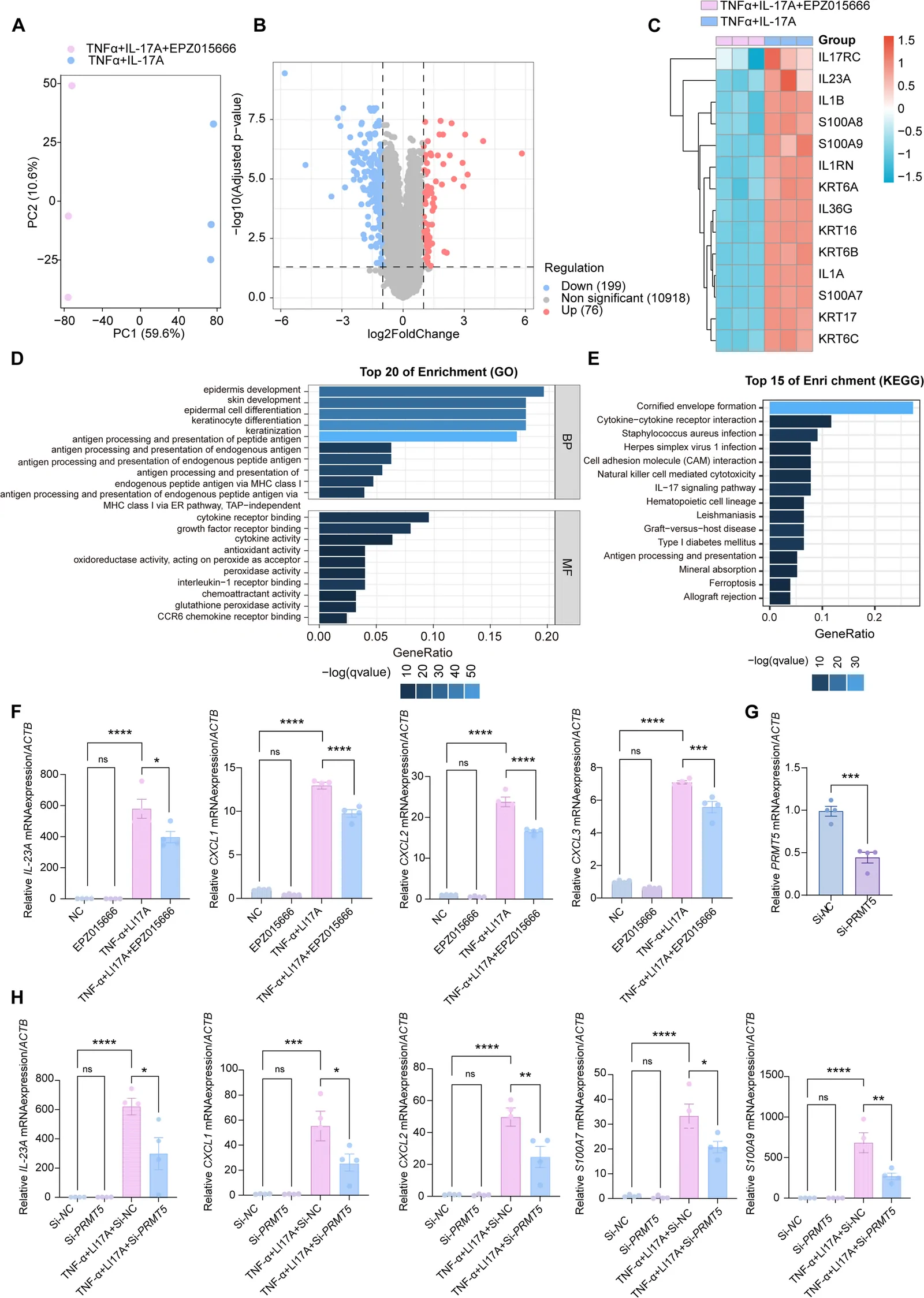
Pharmacological inhibition or knockdown of PRMT5 reduces inflammatory cytokine expression. **A.** PCA plot showing separation between TNFα + IL-17A and TNFα + IL-17A + EPZ015666 treatment groups. **B.** Volcano plot of differentially expressed genes. **C.** Heatmap analysis of inflammatory related differentially expressed genes. **D.** GO analysis of differentially down-regulated pathway between TNFα + IL-17A + EPZ015666 group and TNFα + IL-17A group. **E.** KEGG analysis of differentially down-regulated pathway between TNFα + IL-17A + EPZ015666 group and TNFα + IL-17A group. **F.** qRT-PCR analysis of inflammation-related gene expression in PRMT5 inhibitor -treated keratinocytes. **G**. qRT-PCR validation of PRMT5 expression in human primary keratinocytes transfected with Si-*PRMT5* or Si-*NC*. **H.** qRT-PCR analysis of inflammation-related genes expression in PRMT5 knockdown keratinocytes. Data are presented as mean ± SEM (N = 4 for F–H). Statistical significance was determined by two-tailed unpaired Student’s t test (G) and one-way ANOVA followed by Tukey's multiple comparisons test (F and H). ns, not statistically significant. *p < 0.05, **p < 0.01, ***p < 0.001, ****p < 0.0001

To systematically identify the downstream molecular pathways regulated by PRMT5 in keratinocytes, we performed RNA sequencing on TNF-α- and IL-17A-stimulated primary human keratinocytes treated with EPZ015666 or vehicle control. Principal component analysis (PCA) revealed clear transcriptional separation between the two groups (Fig. 3A), and differential expression analysis identified a substantial number of dysregulated genes in EPZ015666-treated keratinocytes; the downregulated genes were closely linked to psoriasis-related inflammation, as illustrated in the heatmap (Fig. 3B and Fig. 3C). Gene Ontology (GO) and Kyoto Encyclopedia of Genes and Genomes (KEGG) enrichment analyses revealed that downregulated genes were predominantly enriched in inflammatory response pathways, including cytokine–cytokine receptor interaction, IL-17 signaling pathways and cytokine activity, etc. (Fig. 3D and 3E). Notably, the marked suppression of cytokine-related transcriptional programs upon PRMT5 inhibition prompted us to investigate the upstream transcriptional mechanisms involved in inflammatory responses.

### Pharmacological inhibition of PRMT5 and knockdown of *PRMT5* both inhibit inflammatory responses in keratinocytes

To further validate the findings from RNA sequencing and explore how PRMT5 played a role in psoriasis, we treated the human primary keratinocytes with specific PRMT5 inhibitor EPZ015666, with or without TNF-α and IL-17A stimulation, and detected its influence on the reduction of inflammation. The results indicated that EPZ015666 reduced the inflammatory effect (Fig. 3F). To further assess the PRMT5 effects on inflammation, we performed *PRMT5* knockdown in human primary keratinocytes and we also found that *PRMT5* knockdown suppressed TNF-α- and IL-17A-induced inflammatory responses in human primary keratinocytes, markedly reducing the psoriasis related inflammatory factors expression (Fig. 3G and H). To further confirm that the anti-inflammatory effects of EPZ015666 are PRMT5-dependent rather than due to off-target effects, we performed a genetic rescue experiment. Overexpression of *PRMT5* in EPZ015666-treated keratinocytes significantly restored the expression of inflammatory mediators, including *IL23A, CXCL1, and CXCL3,* following TNF-α + IL-17A stimulation (Figure S2), confirming the on-target specificity of EPZ015666. These results directly demonstrate that inhibition of PRMT5 suppresses the keratinocytes inflammation.

### PRMT5 interacts with p65 to promote its symmetric dimethylation and maintain its stability, thereby facilitating inflammatory responses.

**Fig. 4:**
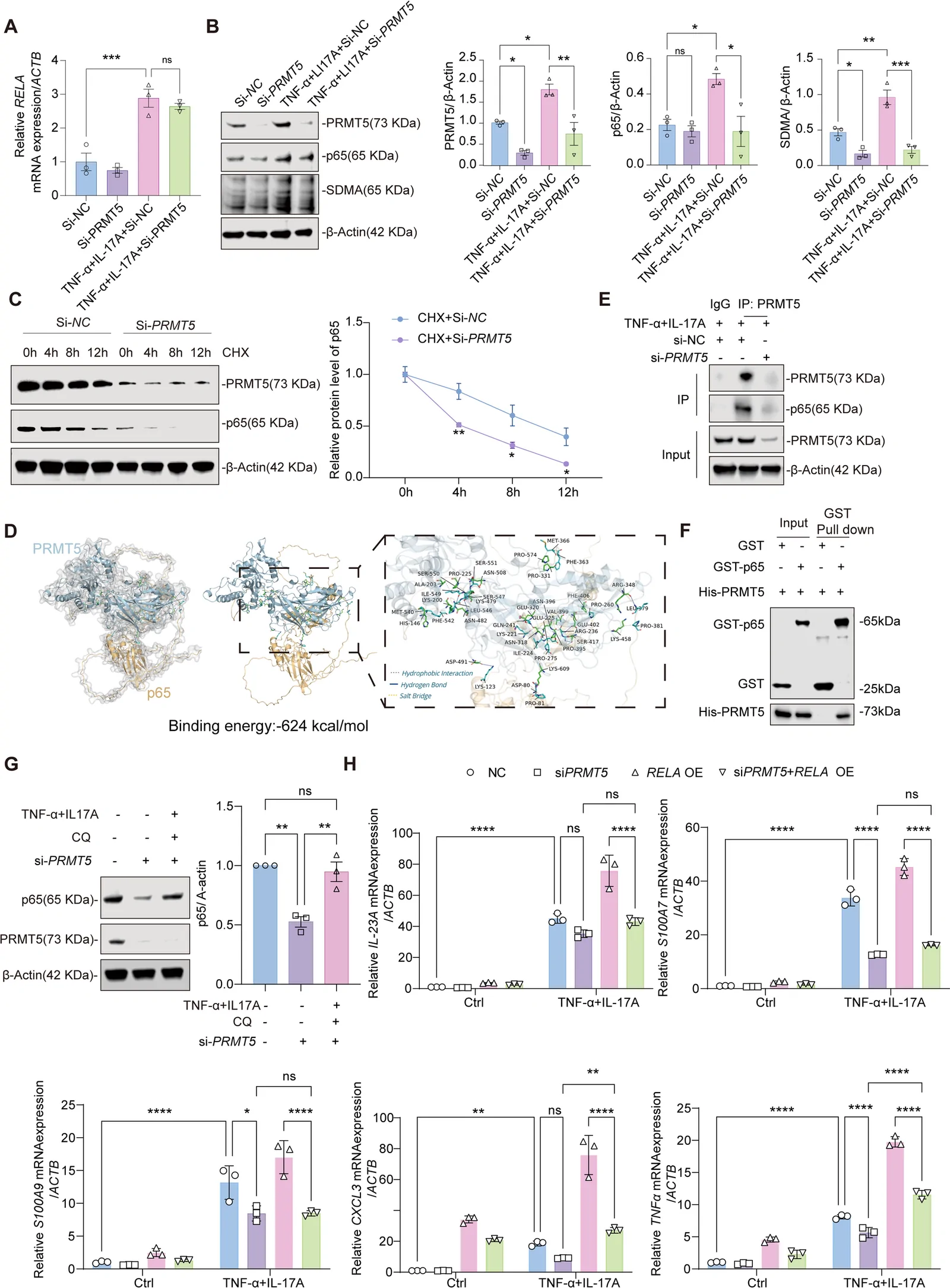
PRMT5 interacts with p65 to promote its symmetric dimethylation and maintain its stability, thereby facilitating inflammatory responses. **A.** qRT-PCR analysis of *RElA* mRNA expression in TNFα + IL-17A -treated primary human keratinocytes transfected with Si-*PRMT5* or Si-*NC*. **B.** Reprensentative immunoblot of PRMT5, p65 and SMDA (Left) and quantification (right) in TNFα + IL-17A -treated primary human keratinocytes transfected with Si-*PRMT5* or Si-*NC*. **C**. Reprensentative immunoblot of PRMT5, p65 (Left) and quantification (right) in CHX (100 μg/ml)-treated primary human keratinocytes transfected with Si-*PRMT5* or Si-*NC*. **D**. Molecular docking analysis illustrating the predicted interaction between PRMT5 and p65. **E.** Reprensentative Co-immunoprecipitation (Co-IP) analysis of the interaction between PRMT5 and p65, performed using anti-PRMT5 antibody in TNFα + IL-17A -treated primary human keratinocytes transfected with Si-*PRMT5* or Si-*NC*. **F**. GST pull-down assay showing the interaction between p65 and PRMT5. **G** Reprensentative immunoblot of p65 protein level in keratinocytes treated with si-*PRMT5* in the presence or absence of the lysosomal inhibitor chloroquine (CQ) under TNF-α + IL-17A stimulation. **H.** qRT-PCR analysis of inflammation-related gene expression in a rescue assay in which Si-*PRMT5*-transfected primary human keratinocytes were co-transfected with *RELA* overexpression. Data are presented as mean ± SEM (N = 3 for A-C, G and H). Statistical significance was determined by two-way (C) and one-way ANOVA followed by Tukey's multiple comparisons test (A-B, G and H). ns, not statistically significant. *p < 0.05, **p < 0.01, ***p < 0.001, ****p < 0.0001.

Given that p65 is a master transcriptional regulator of inflammatory cytokine expression in keratinocytes, we investigated the functional relationship between PRMT5 and p65. Western blot analysis revealed that siRNA-mediated *PRMT5* knockdown significantly reduced p65 protein levels without affecting *RELA* mRNA expression. This was accompanied by a reduction in global SDMA levels, suggesting that PRMT5 regulates p65 post-translationally (Fig. 4A and 4B). Similarly, pharmacological inhibition of PRMT5 with EPZ015666 significantly reduced SDMA levels at 65 kDa and p65 protein expression (Figure S3A), confirming on-target inhibition of PRMT5-mediated p65 methylation. To directly assess whether PRMT5 affects p65 protein stability, we treated keratinocytes with the protein synthesis inhibitor cycloheximide (CHX) to block de novo protein production and isolate PRMT5-dependent effects on p65 stability. Under conditions of blocked protein synthesis, *PRMT5* knockdown markedly accelerated p65 degradation, confirming that PRMT5 maintains p65 stability at the post-translational level. (Fig. 4C).

To determine whether PRMT5 and p65 interact directly, we performed molecular docking analysis using GRAMM, which predicted a direct binding interaction with an energy of −624 kcal/mol. PLIP and PyMOL analysis of the binding interface identified 12 hydrogen bonds, 13 hydrophobic interactions, and 2 salt bridges (Fig. 4D), collectively indicative of a stable and specific complex. Co-immunoprecipitation assays in TNF-α- and IL-17A-stimulated primary keratinocytes further confirmed a direct endogenous interaction between PRMT5 and p65, which was markedly attenuated upon *PRMT5* knockdown (Fig. 4E). GST pull-down assay further confirmed the direct binding of PRMT5 and p65 (Fig. 4F). To investigate the mechanism by which PRMT5 regulates p65 protein stability, we examined two major protein degradation pathways. Treatment with the lysosomal inhibitor chloroquine (CQ) but not proteasome inhibitor MG132 significantly restored p65 protein levels upon *PRMT5* knockdown under TNF-α + IL-17A stimulation (Fig. 4G and Fig.S4A), demonstrating that PRMT5 protects p65 from lysosomal degradation. Functionally, overexpression of *RELA* in PRMT5-knockdown keratinocytes restored pro-inflammatory cytokine expression to levels comparable to controls, confirming that PRMT5 sustains keratinocyte inflammation through the p65 axis (Fig. 4H). Furthermore, to determine whether the anti-inflammatory effects of EPZ015666 are specifically mediated through PRMT5-p65 methylation rather than broader signaling interference, we assessed both p65 SDMA levels and the activation status of STAT3 and MAPK signaling pathways. The results revealed that EPZ015666 had no significant effect on the phosphorylation of STAT3, ERK, JNK, or P38 under either basal or inflammatory conditions (Figure S3B), demonstrating that EPZ015666 does not broadly suppress upstream inflammatory kinase cascades.

Collectively, these data demonstrate that PRMT5 specifically and directly interacts with p65, maintains its protein stability, and thereby sustains NF-κB signaling activity in psoriatic keratinocytes.

### PRMT5 mediates symmetric dimethylation of p65 at R158 and R166 to enhance NF-κB-mediated inflammatory responses

**Fig. 5:**
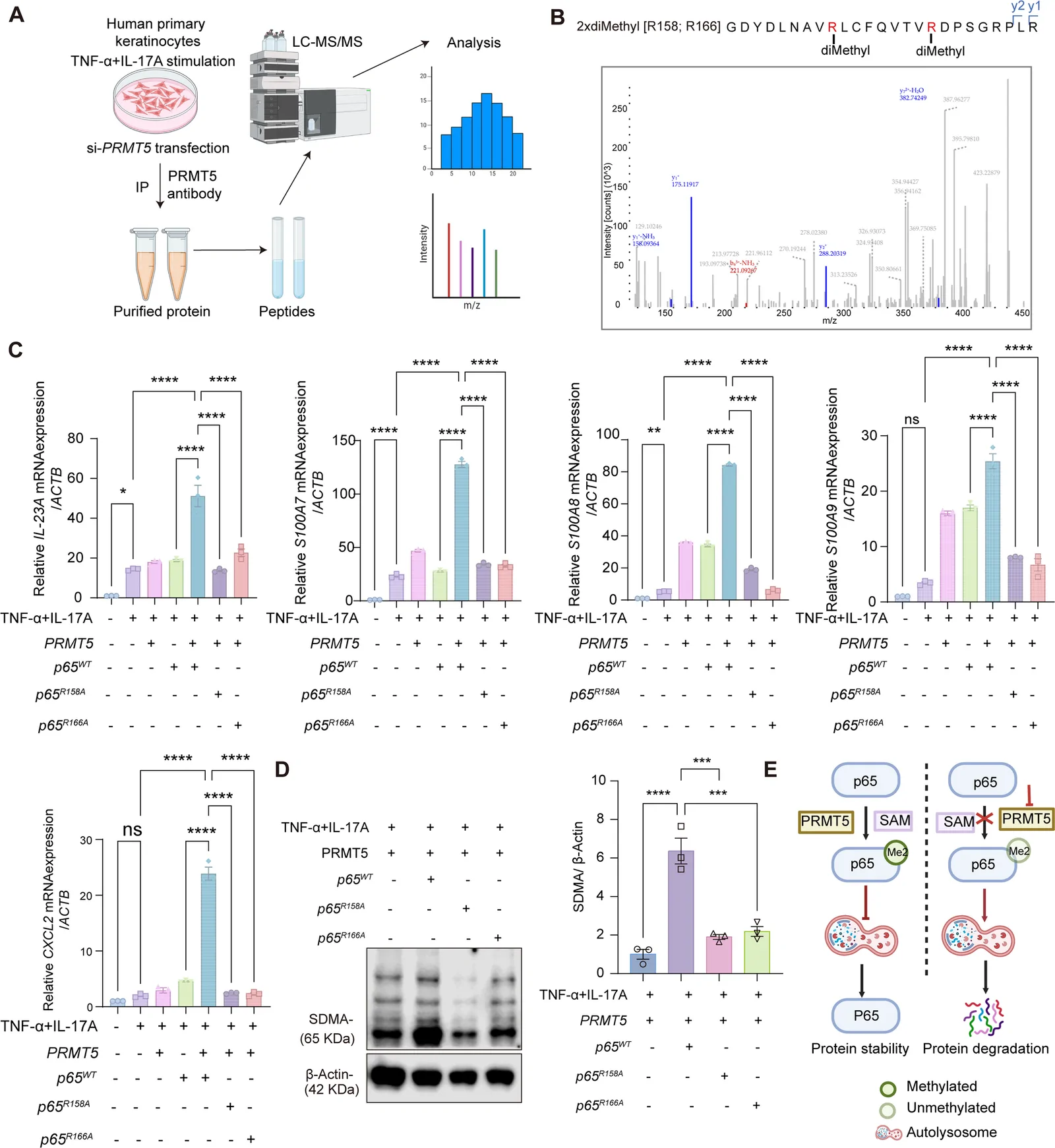
PRMT5 symmetrically dimethylates p65 at R158 and R166 to sustain p65-dependent inflammatory gene expression in keratinocytes. **A** Schematic diagram of the experimental workflow: PRMT5 antibody immunoprecipitated cell lysate from PRMT5-transfected human primary keratinocyte cells under TNF-α and IL17A treatment and subsequent liquid chromatography-tandem mass spectrometry (LC–MS/MS) analysis. **B** Representative LC–MS/MS spectra identifying the methylation sites of p65 at arginine residues R158 and R166. C qRT-PCR analysis of inflammation-related genes expression in primary human keratinocytes co-transfected with PRMT5 and either wild-type P65 (P65^WT^), P65^R158A^, or P65^R166A^.. **D** Western blot analysis of SDMA levels at 65 kDa in keratinocytes co-transfected with PRMT5 and p65 ^WT^, p65^R158A^ or p65^R166A^ under TNF-α + IL-17A stimulation. **E** Schematic illustration of the proposed mechanism by which PRMT5 promotes p65 protein stability: PRMT5 transfers two methyl groups from the SAM (S-adenosylmethionine) methyl donor to the arginine residues of p65, resulting in symmetric dimethylation that enhances p65 stability and sustains its driven inflammatory signaling. Data are presented as mean ± SEM (N = 3 for C-D). Statistical significance was determined by one-way ANOVA followed by Tukey's multiple comparisons test (C-D). Exact p-values are indicated in the figure

Having established that PRMT5 interacts with and stabilizes p65, we next determined whether PRMT5 modifies p65 through symmetric arginine dimethylation (SDMA). LC–MS/MS analysis of PRMT5 immunoprecipitates from TNF-α and IL-17A-stimulated keratinocytes identified symmetric dimethylation at R158, R166, and R336 of p65 (Fig. 5A, 5B and Fig.S5A). To assess the functional significance of each site, we generated alanine-substitution mutants at each position (p65 ^R158A^, p65 ^R166A^ and p65 ^R336A^). In rescue experiments, overexpression of wild-type p65 in *PRMT5*-depleted keratinocytes restored pro-inflammatory cytokine production; by contrast, overexpression of the p65 ^R158A^, p65 ^R166A^ but not p65 ^R336A^, failed to rescue cytokine production even upon concurrent *PRMT5* overexpression (Fig. 5C, and Fig. S5B-C), which is correlated with p65 protein level(Fig.S5D). Of note, both p65 ^R158A^ and p65 ^R166A^ mutations significantly reduced SDMA levels compared with p65^WT^, indicating that R158 and R166 are the key arginine residues methylated by PRMT5 (Fig.5D). These findings establish a PRMT5–p65 regulatory axis in which symmetric dimethylation at R158 and R166 stabilizes p65 by preventing lysosomal degradation, thereby sustaining NF-κB transcriptional activity in inflammatory keratinocytes (Fig. 5E).

### PRMT5 inhibition alleviates psoriatic skin inflammation and immune cell infiltration in vivo

**Fig. 6:**
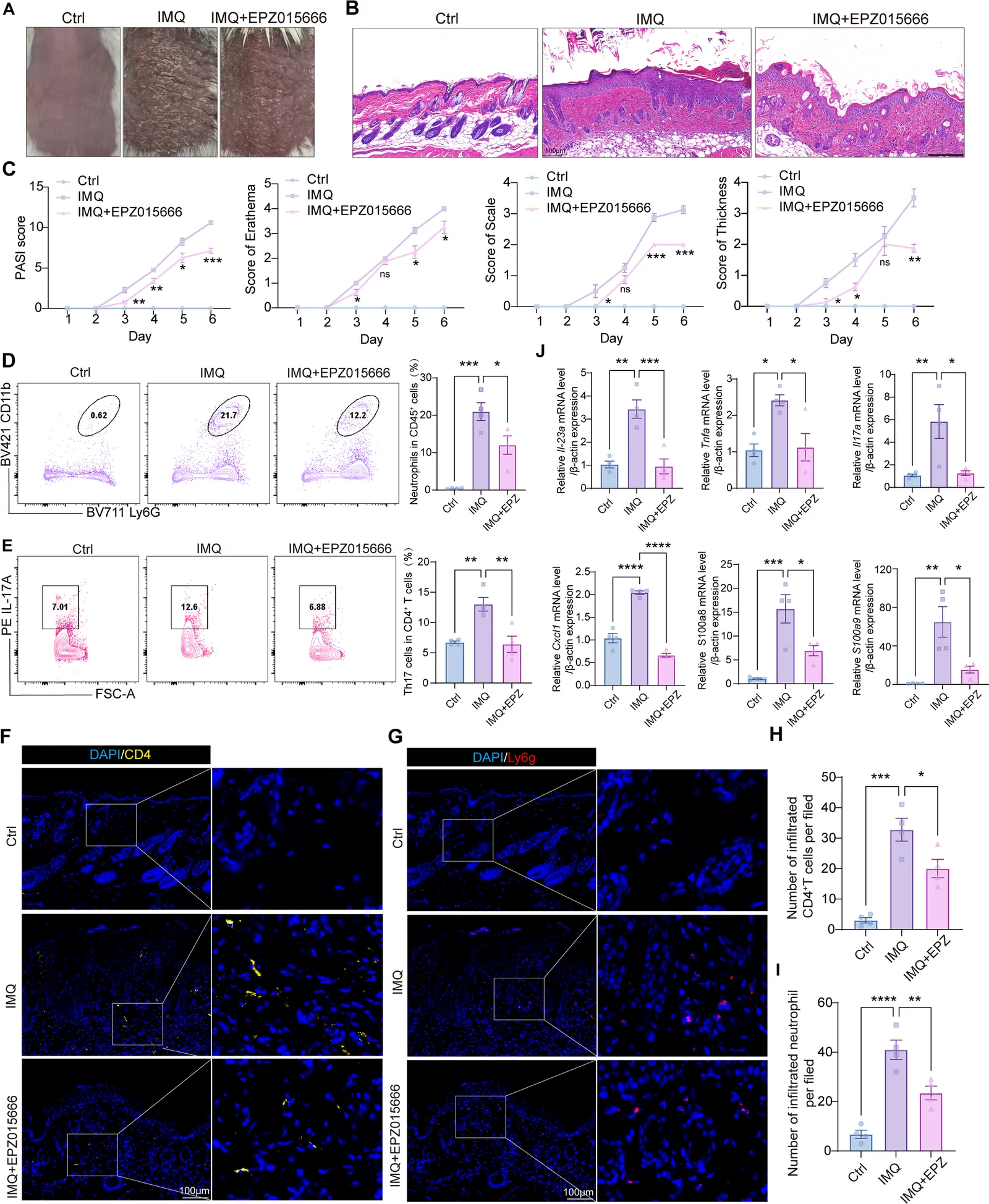
Pharmacological inhibition of PRMT5 alleviates psoriasis-like inflammation responses. **A** Gross morphology of mouse dorsal skin treated with IMQ, combined with EPZ015666 or vehicle. **B** H&E staining of skin sections from the four treatment groups illustrating epidermal changes (scale bar = 100 μm). **C** Time-course clinical scores (erythema, scale, thickness, and PASI) over 6 days across treatment groups. **D**. The representative image (left panel) and the percentage (right panel) of neutrophils among infiltrating immune cells in lesional skin, assessed by flow cytometry. **E** The representative image (left panel) and the percentage (right panel) of Th17 cells among infiltrating immune cells in lesional skin, assessed by flow cytometry. **F** Immunofluorescence staining for CD4 in mouse dorsal skin treated with IMQ, combined with EPZ015666 or vehicle. **G** Immunofluorescence staining for Ly6G in mouse dorsal skin treated with IMQ, combined with EPZ015666 or vehicle. **H** Quantification of CD4^+^T cells number of F. **I** Quantification of neutrophils number of G. Data are presented as mean ± SEM (N = 4 for C-E and H-I). Statistical significance was determined by two-way (C) or one-way ANOVA followed by Tukey's multiple comparisons test (D, E and H-I). ns, not statistically significant. *p < 0.05, **p < 0.01, ***p < 0.001, ****p < 0.0001.

To evaluate the translational potential of PRMT5 inhibition, we administered EPZ015666 systemically in the IMQ-induced murine psoriasis model. EPZ015666 treatment significantly attenuated these psoriasis-like manifestations, including erythema, scaling, and dorsal skin thickness (Fig. 6A and 6 C). H&E staining confirmed that prominent epidermal hyperplasia observed in IMQ-treated mice was markedly reduced in the EPZ015666 group (Fig. 6B). Flow cytometric analysis of lesional skin demonstrated significant reductions in both neutrophil and Th17 cell infiltration following EPZ015666 treatment (Fig. 6D and 6E). Additionally, immunofluorescence staining further confirmed reductions in CD4^+^ T cells and neutrophil infiltration (Fig. 6F-I). Correspondingly, the expression of psoriasis-associated inflammatory markers-including *Il-17a, Il23, Tnfα, Cxcl1, S100a8, and S100a9*-was significantly downregulated in EPZ015666-treated mice relative to the IMQ group (Fig. 6J). Consistently, EPZ015666 treatment also significantly reduced IL-23-induced epidermal hyperplasia and skin erythema in an independent murine psoriasis model, as confirmed by gross morphology, H&E staining, and epidermal thickness quantification (Fig. S6 A-C). These in *vivo* findings establish that pharmacological targeting of PRMT5 effectively suppresses psoriatic inflammation, supporting its translational potential as a therapeutic strategy for psoriasis.

## Discussion

Psoriasis is a prevalent chronic inflammatory skin condition driven by dysregulated immune activity and abnormal keratinocyte function. Keratinocytes play essential roles in both the initiation and maintenance phases of psoriasis. As key components of the innate immune defense, keratinocytes are capable of sensing diverse triggers and mounting robust responses through the secretion of an extensive array of chemokines, antimicrobial peptides, and pro-inflammatory mediators. These signals coordinate the recruitment and activation of infiltrating immune cells, ultimately fostering and sustaining the self-reinforcing inflammatory loop that underlies the pathogenesis of psoriasis. In this study, we identified the protein arginine methyltransferase PRMT5 as significantly upregulated in psoriatic keratinocytes and contributed to the disease pathogenesis. Mechanistically, PRMT5 physically interacts with p65 and symmetrically dimethylates it at arginine residues R158 and R166, thereby stabilizing p65 protein, promoting cytokine secretion, and ultimately driving immune cell infiltration. Finally, pharmacological inhibition of PRMT5 with EPZ015666 ameliorates psoriatic inflammatory responses, suggesting its potential as a therapeutic target for regulating inflammatory responses in psoriasis.

As a type II arginine methyltransferase, PRMT5 catalyzes the SDMA on protein arginine and has garnered attention for its role in influencing chromatin remodeling, transcriptional repression, RNA splicing, and intracellular signaling (Alipourgivi et al. 2025). Aberrant overexpression or hyperactivation of PRMT5 has been documented across a broad spectrum of malignancies, including glioblastoma and lung, pancreatic, colorectal, prostate, and ovarian cancers (Prabhu 2022; Ozturk 2025; Zhou 2023). It has become an attractive therapeutic target for cancer. Besides, studies have also revealed that PRMT5 can act as an on–off switch for endothelial cell inflammation, suggesting a role for PRMT5 beyond oncology, extending to inflammatory disease contexts. However, the role of PRMT5 in the keratinocyte inflammatory response and skin inflammatory disease such as psoriasis remains unclear. Our study identifies PRMT5 as a potent driver of inflammation in the context of psoriasis. This role is substantiated by both genetic ablation (*Prmt5*deficiency) and pharmacological inhibition experiments, each of which demonstrates a marked attenuation of inflammatory responses upon PRMT5 inhibition. Collectively, these findings suggest that targeting PRMT5 in keratinocytes to restrain aberrant inflammatory signaling represents a promising therapeutic strategy for psoriasis. Currently, PRMT5 inhibitors have been advanced into clinical evaluation across multiple oncological settings. AMG 193, a CNS-penetrant MTA-cooperative PRMT5 inhibitor, demonstrated a favorable safety profile devoid of clinically significant myelosuppression in patients with MTAP-deleted solid tumors, achieving an objective response rate of 21.4% across eight distinct tumor types in a phase I dose-exploration study (NCT03975712) (Rodon 2024). More recently, the first-generation PRMT5 inhibitor GSK3326595 also showed preliminary clinical activity in patients with solid tumors and non-Hodgkin lymphoma (Gounder 2025). The manageable safety profiles observed with these agents suggest that pharmacological PRMT5 inhibition may be suitable for chronic administration in non-oncological conditions. Our study indicates that inhibition of PRMT5 in keratinocytes attenuates aberrant inflammatory signaling, offering a theoretical basis for the development of targeting PRMT5.

As a key transcription factor in the NF-κB signaling pathway, p65 (RelA) is responsible for regulating cellular responses to a variety of inflammatory cytokines and immune stimuli (Sun 2024). Its diverse functions are central to the pathogenesis of psoriasis, where NF-κB signaling has been shown to alter the behaviors of keratinocytes and immune cells by affecting their proliferation, differentiation, and production of cytokines and chemokines, and where NF-κB-related pathway activity has further been associated with patient clinical outcomes (Guo 2023; Mao et al. 2025). Post-translational modifications of the p65 subunit, such as phosphorylation, acetylation, and ubiquitination, have been extensively characterized as regulatory mechanisms of NF-κB transcriptional activity (Yang 2021; Wang et al. 2019; Thatikonda et al. 2020), the contribution of arginine methylation to psoriatic inflammation remains poorly understood. Protein methylation has also emerged as an important regulatory mechanism of p65 function. PRMT5 has been previously demonstrated to regulate NF-κB activity through methylation of the p65 subunit: Wei et al. demonstrated that methylation of p65 at Arg30 by PRMT5 in response to IL-1β stimulation promotes the ability of NF-κB to bind to kB elements for gene transcription (Wei 2013). Furthermore, R35, R174, R304, and R330 in addition to R30, have been reported to be methylated by PRMT5, with methylation at both sites participating in the transcription of a subset of TNF-α-induced pro-inflammatory genes, most notably CXCL10- in endothelial cells (Harris et al. 2014, 2016). Collectively, the emerging evidence indicates that PRMT5-mediated arginine methylation of p65 is highly site-specific, with individual methylation events responding to distinct upstream inflammatory stimuli and conferring selectivity over discrete sets of downstream target genes. However, whether such arginine methylation events occur in keratinocytes in the context of psoriasis, and whether p65 protein stability is similarly influenced by methylation in this setting, remains unclear. In the present study, we identify a previously unreported modification: PRMT5-mediated symmetric dimethylation of p65 at Arg^158^ and Arg^166^ (hereafter referred to as p65-R158/R166me2s), and demonstrate its functional importance in NF-κB-dependent transcription of psoriasis-associated cytokines. Notably, we further demonstrate that this dimethylation protects p65 from autophagy-mediated lysosomal degradation, thereby maintaining p65 protein stability and sustaining NF-κB transcriptional activity in keratinocytes.

Several limitations should be acknowledged. First, neither the IMQ nor the IL-23 murine model fully recapitulates the chronicity and immunological complexity of human psoriasis, and findings from these models should therefore be interpreted with caution. Second, although we identified PRMT5-mediated symmetric dimethylation of p65 at R158 and R166 as a critical regulatory modification, our study focused predominantly on keratinocyte-intrinsic mechanisms, and the extent to which PRMT5 may similarly regulate p65 activity in other psoriasis-relevant cell types-including dendritic cells and Th17 cells that drive the IL-23/IL-17 inflammatory axis (Fitch et al. 2007) remains unexplored. Third, while EPZ015666 demonstrated promising anti-inflammatory efficacy in both murine models, its direct clinical applicability remains to be established, given that its long-term safety profile, tissue bioavailability, and suitability for topical or systemic delivery in chronic skin disease have not been characterized. Future studies employing patient-derived skin organoids or ex vivo human psoriatic tissue will be essential to bridge the gap between our findings and potential clinical application.

In conclusion, our findings unveil a novel regulatory axis in which PRMT5 sustains keratinocyte-driven inflammation in psoriasis by methylating p65 at R158 and R166, thereby suppressing autophagy-dependent lysosomal degradation and maintaining NF-κB transcriptional activity.

## Supplementary Information

Supplementary Material 1.

Supplementary Material 2.

## Data Availability

The RNA-seq raw data (FASTQ files) and associated metadata supporting the findings of this study have been deposited in the NCBI Sequence Read Archive (SRA) underthe following BioProject accessions: RNA-seq (PRJNA1453462). The mass spectrometry proteomics data have been deposited to the ProteomeXchange Consortium via the iProX partner repository with the dataset identifier PXD077294 and project identifier IPX0016713000. These datasets will be made publicly available upon publication of this article. The datasets used and analyzed during the current study are available from the corresponding author on reasonable request.

## Abbreviations

PRMT5: Protein arginine methyltransferase 5
SDMA: Symmetric dimethylarginine
ADMA: Asymmetric dimethylarginine
PASI: Psoriasis Area and Severity Index
RNA-seq: RNA-sequencing
scRNA-seq: Single-cell RNA-sequencing
DEGs: Differentially expressed genes
PCA: Principal component analysis
Pan-CK: Pan-cytokeratin
IMQ: Imiquimod
GEO: Gene Expression Omnibus
HC: Healthy controls
PN: Psoriasis non-lesional
PL: Psoriasis lesional
KGM: Keratinocyte Growth Medium
OD: Optical density
CHX: Cycloheximide
