# Supplementary material for: PRMT5 promotes psoriatic inflammation through mediating dimethylation of p65 in keratinocytes
Source: Mol Med. 2026 Jul 10;32:172. doi: 10.1186/s10020-026-01558-6 (PMC13637110; doi:10.1186/s10020-026-01558-6)

Fig1H

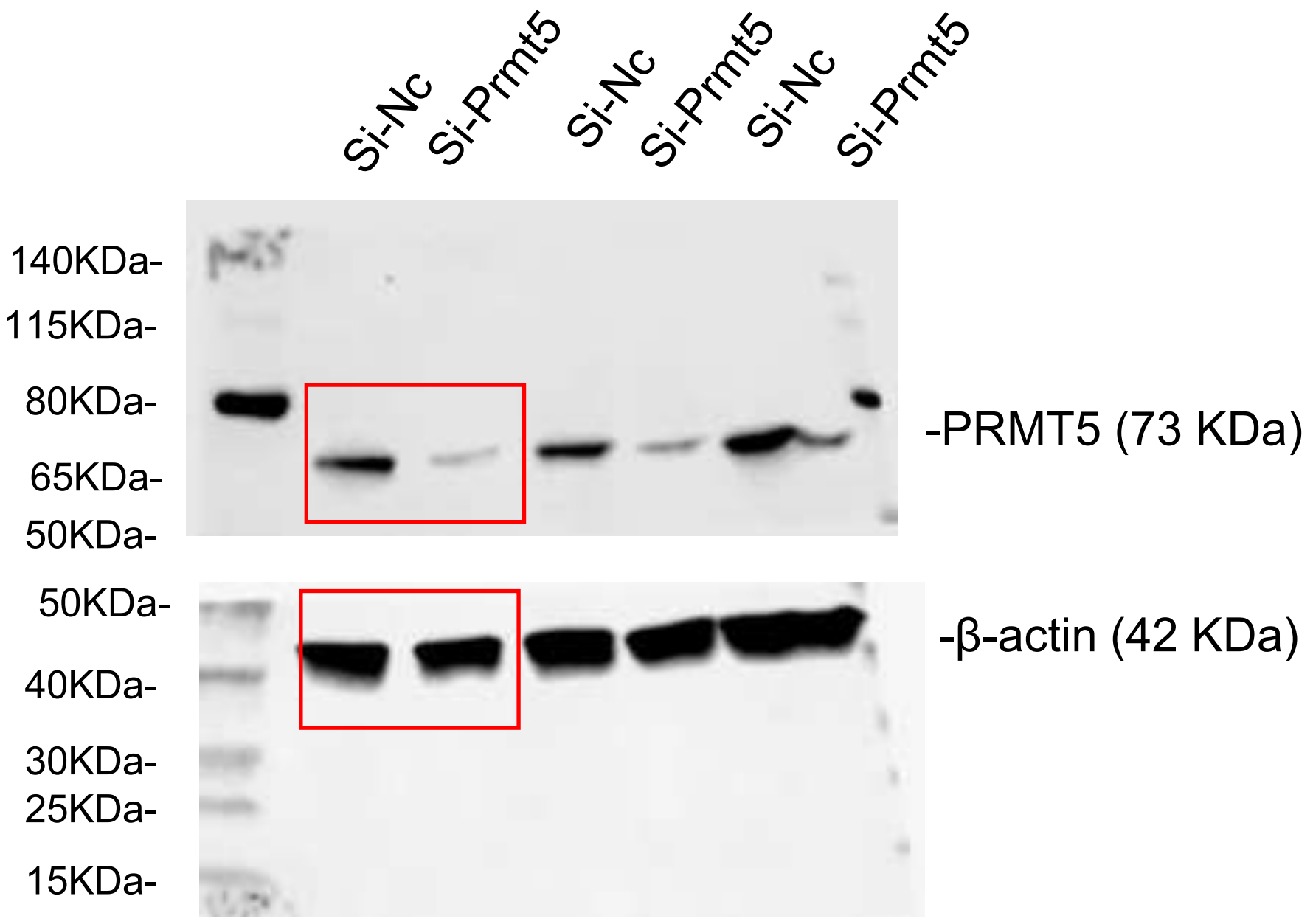

Thermo Scientific PageRuler Protein Marker
26616 in 4-12% Bis-Tris Gel

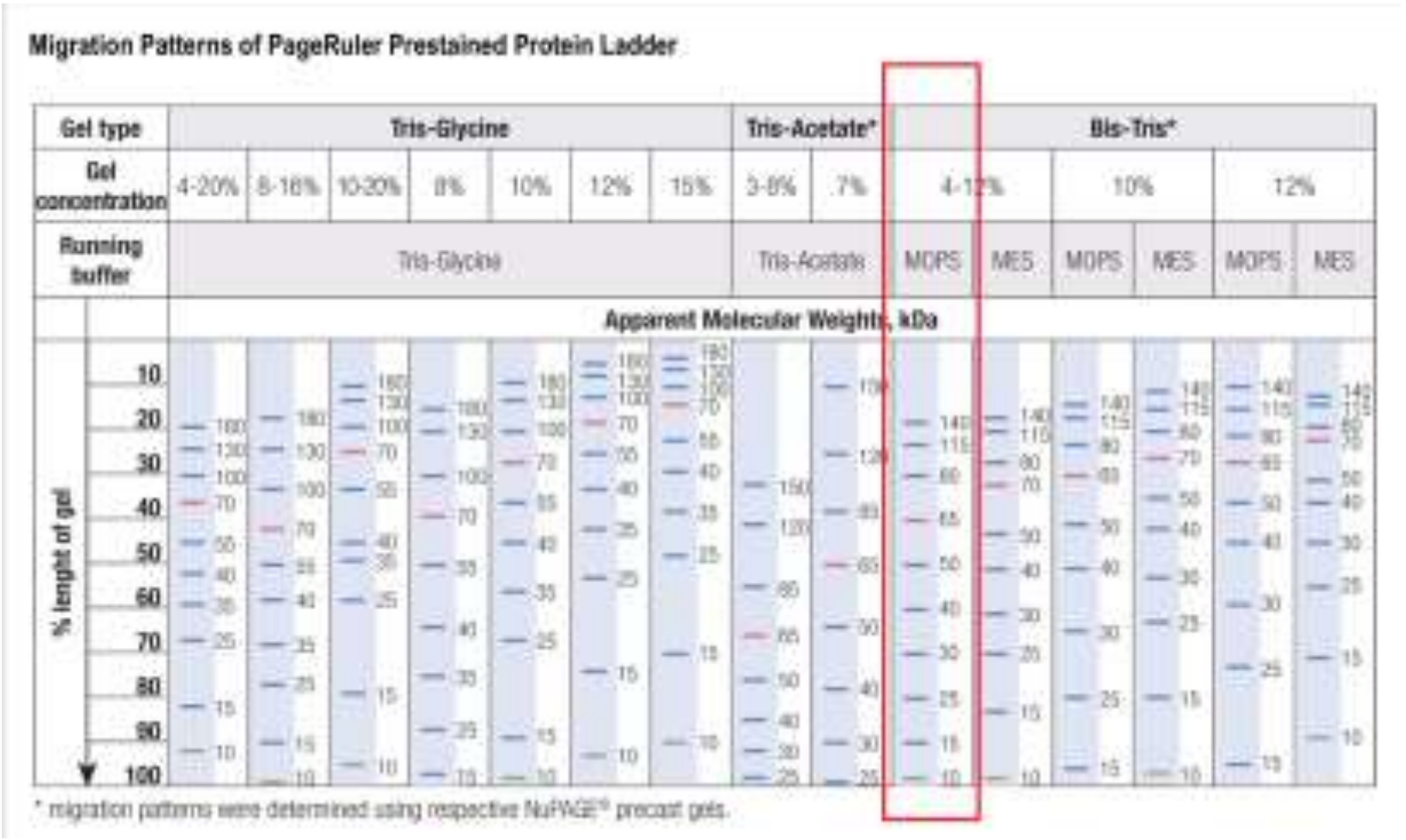

Fig4B

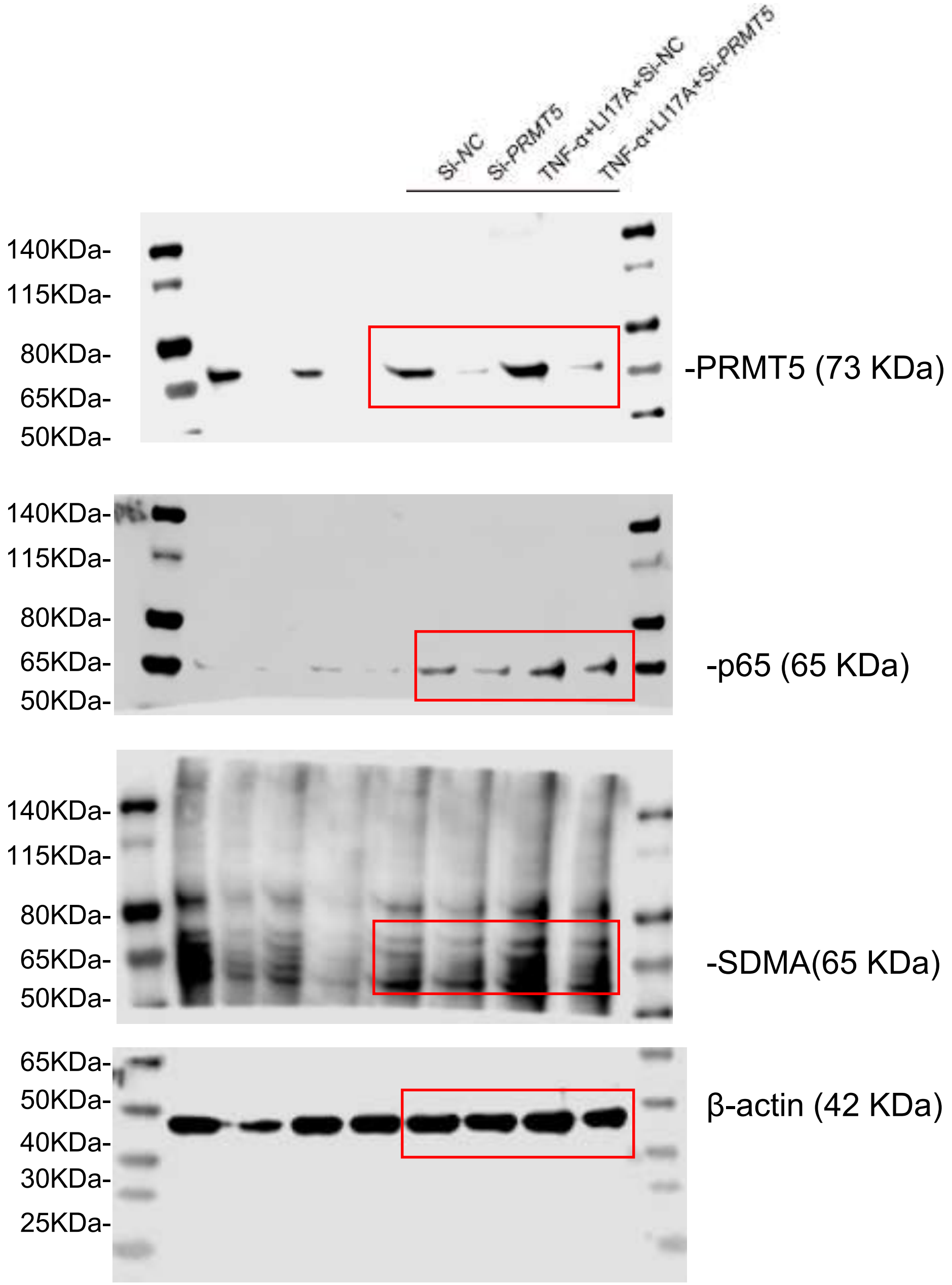

Thermo Scientific PageRuler Protein Marker 26616 in 4-12% Bis-Tris Gel

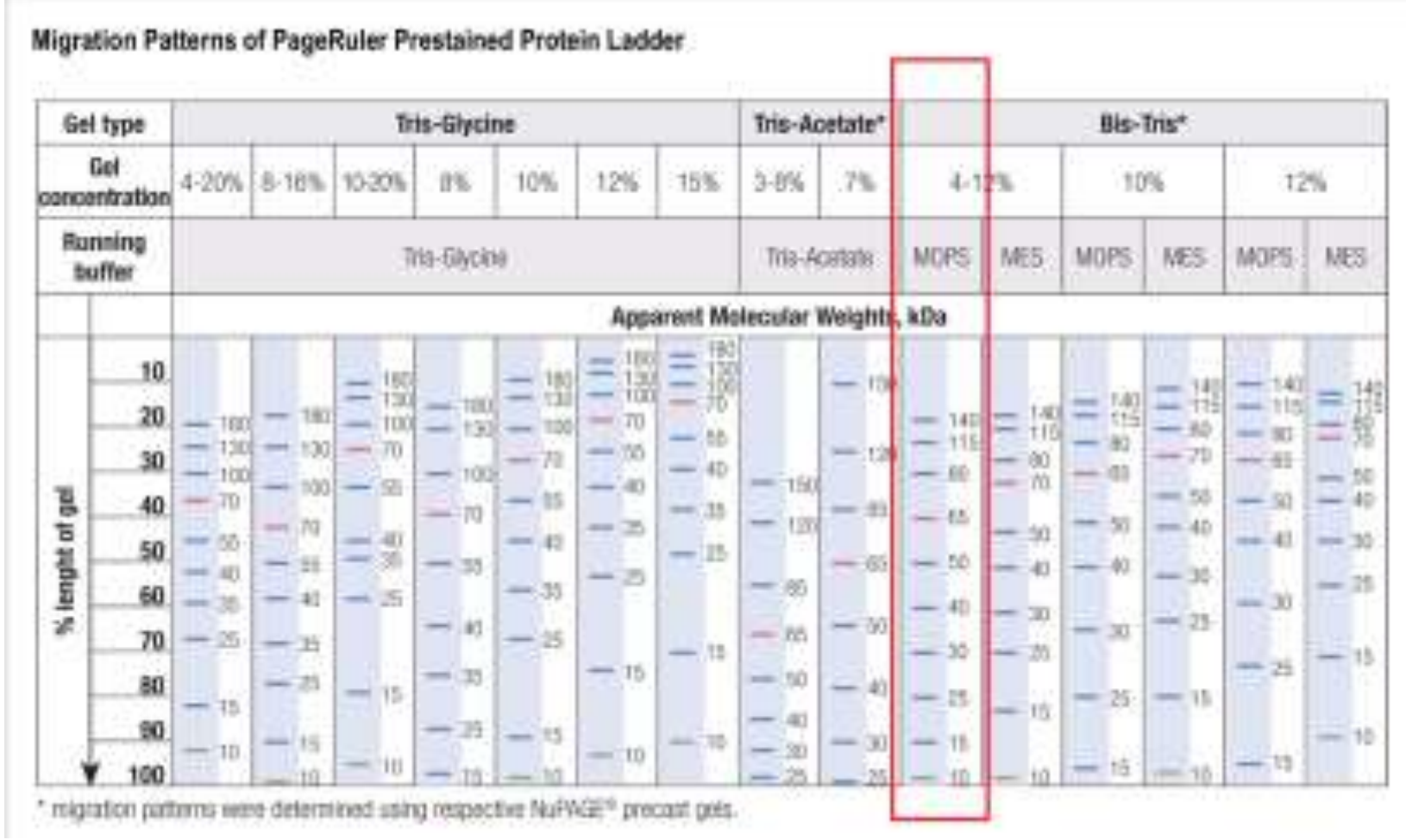

Fig4C

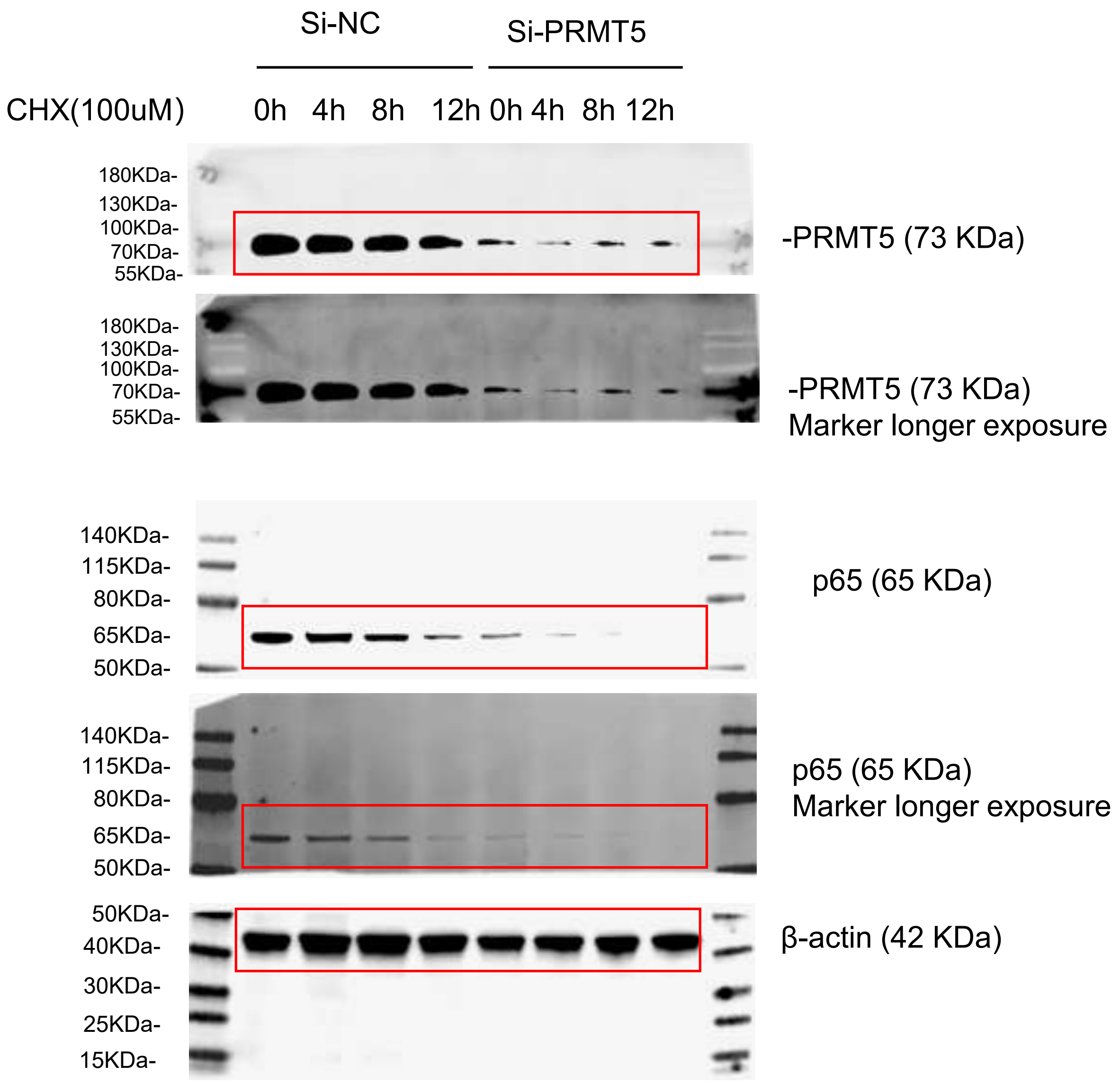

PRMT5
Thermo Scientific PageRuler Protein Marker
#26616 in 10% Tris-Glycine Gel

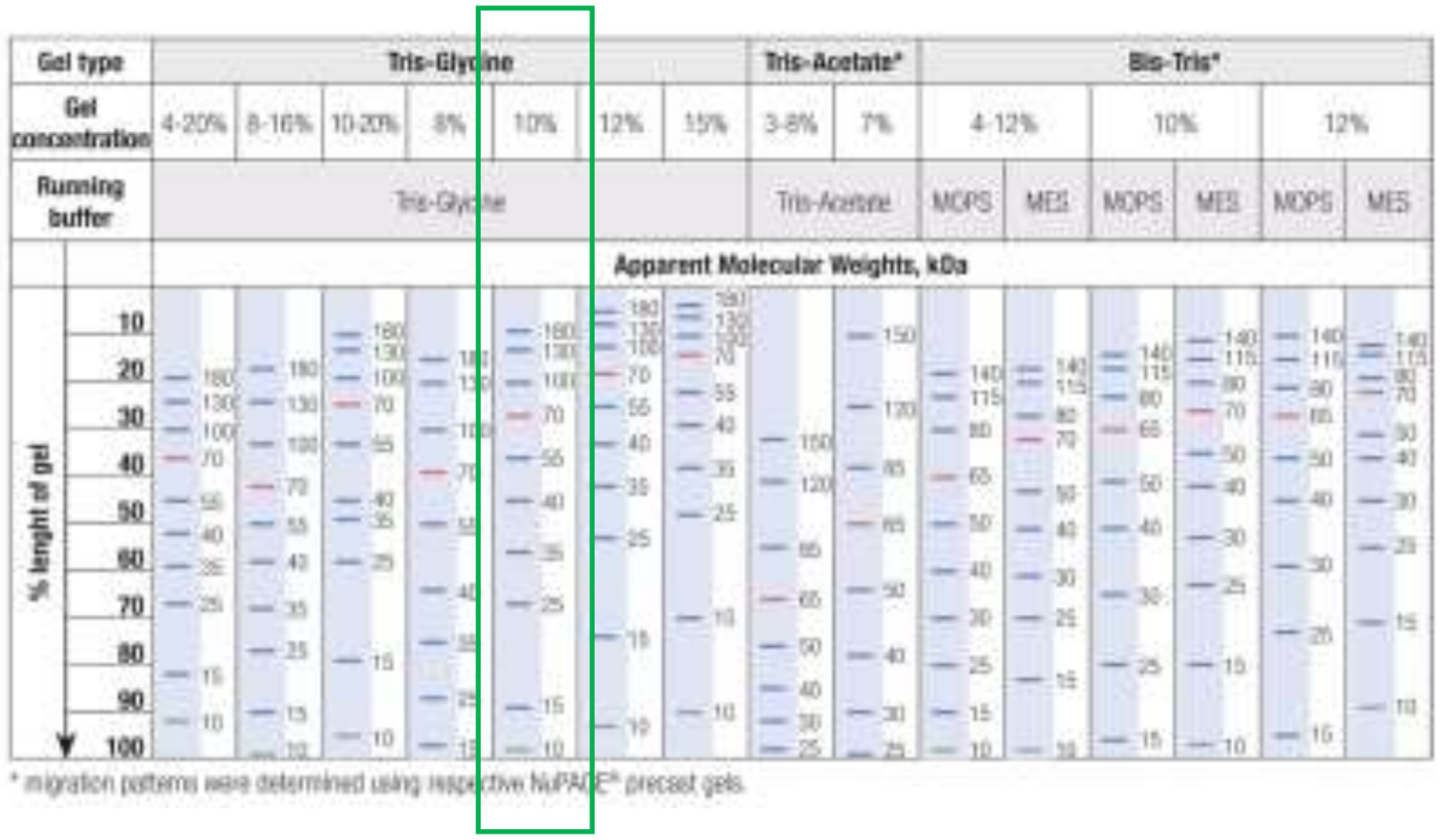

P65，β-actin
Thermo Scientific PageRuler Protein Marker
#26616 in 4-12% Bis-Tris Gel

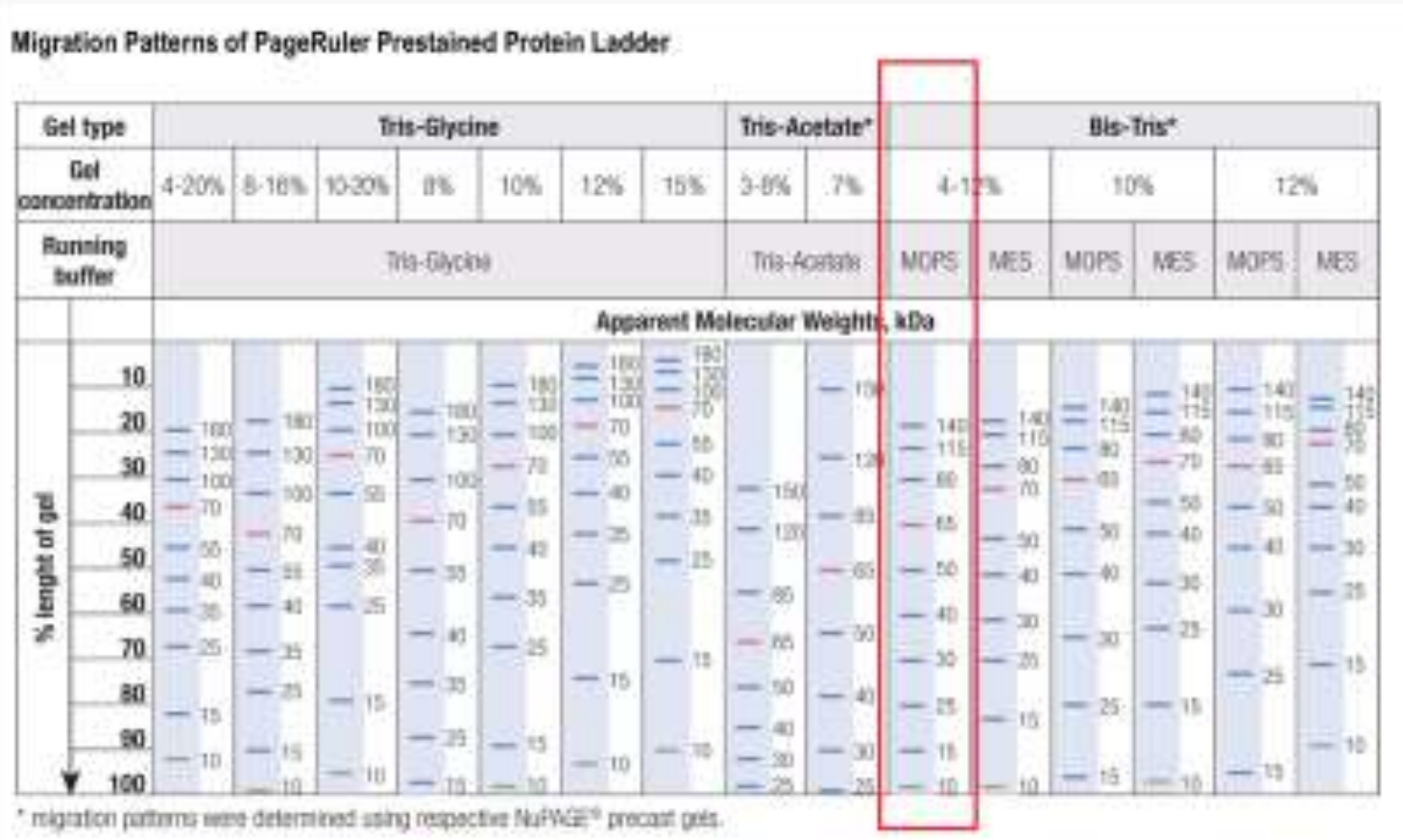

Fig4E

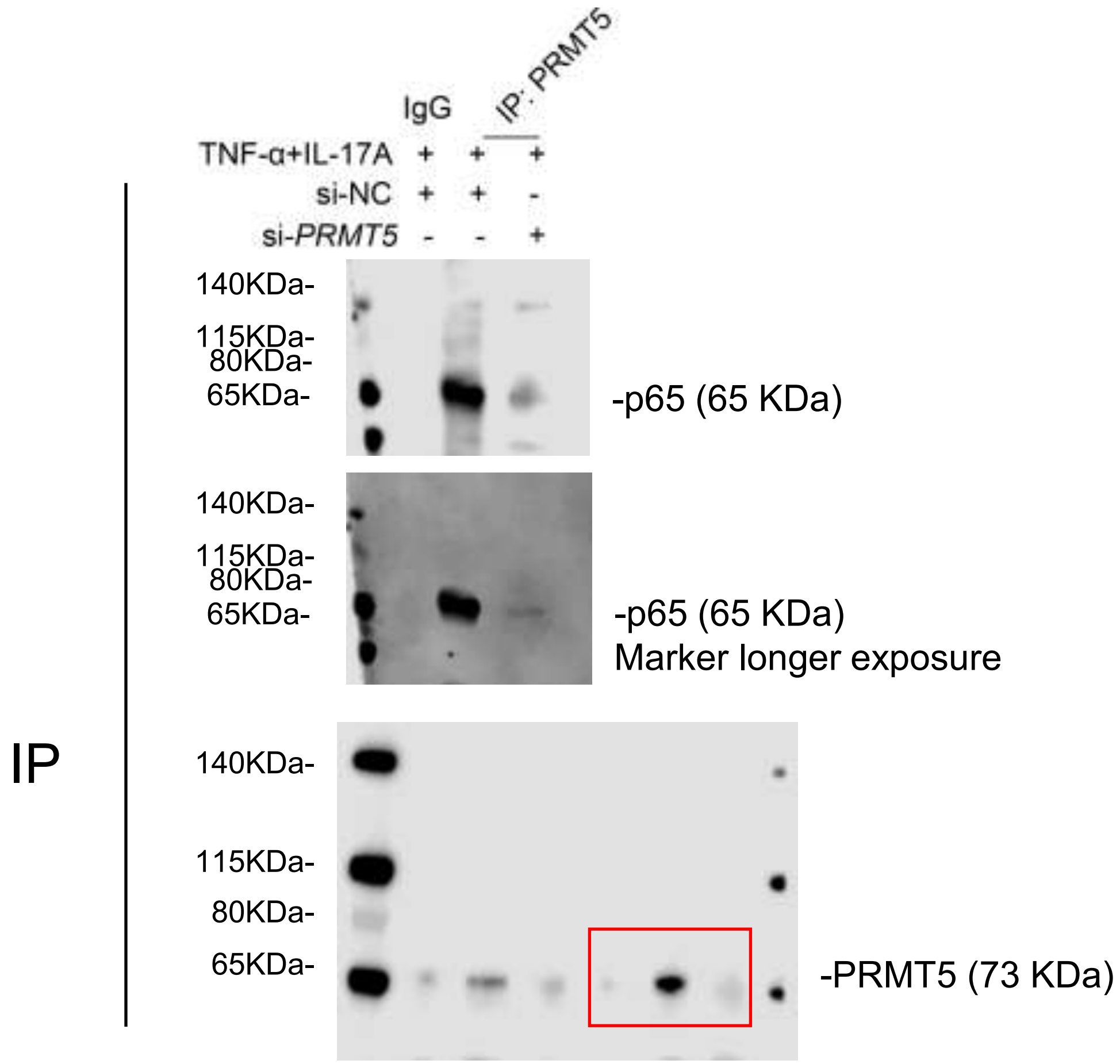

Thermo Scientific PageRuler Protein Marker
#26616 in 4-12% Bis-Tris Gel

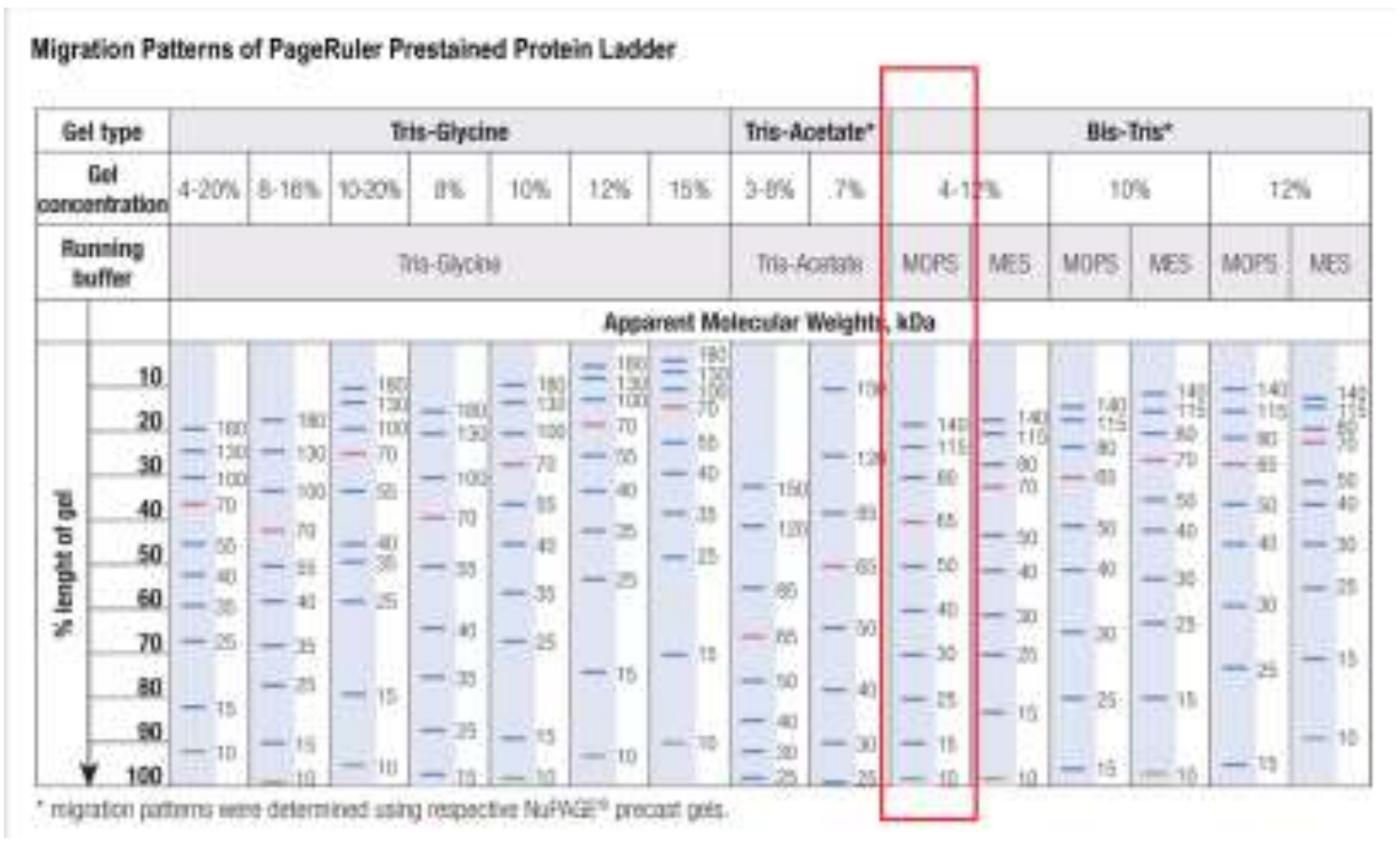

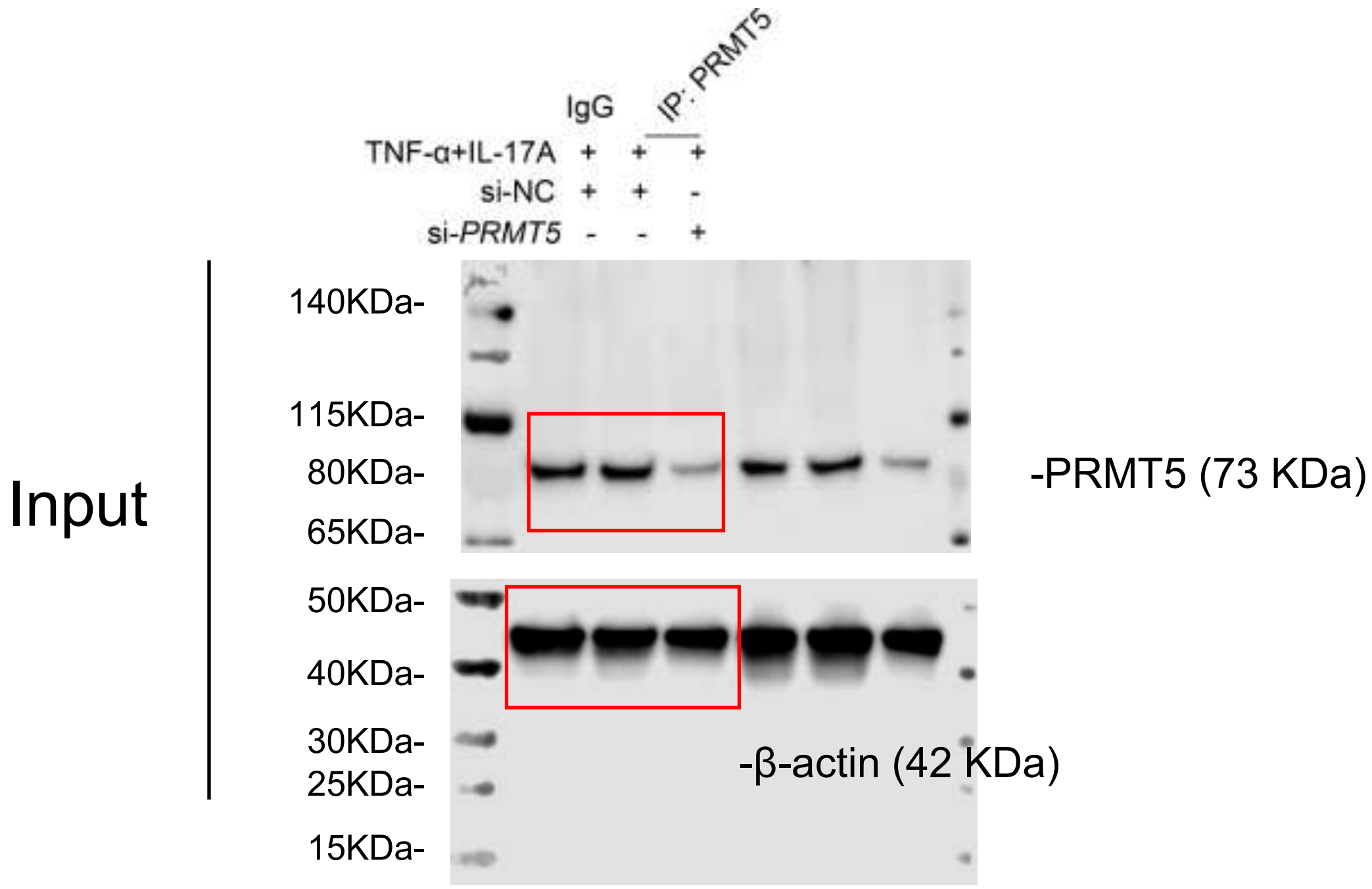

Fig4F

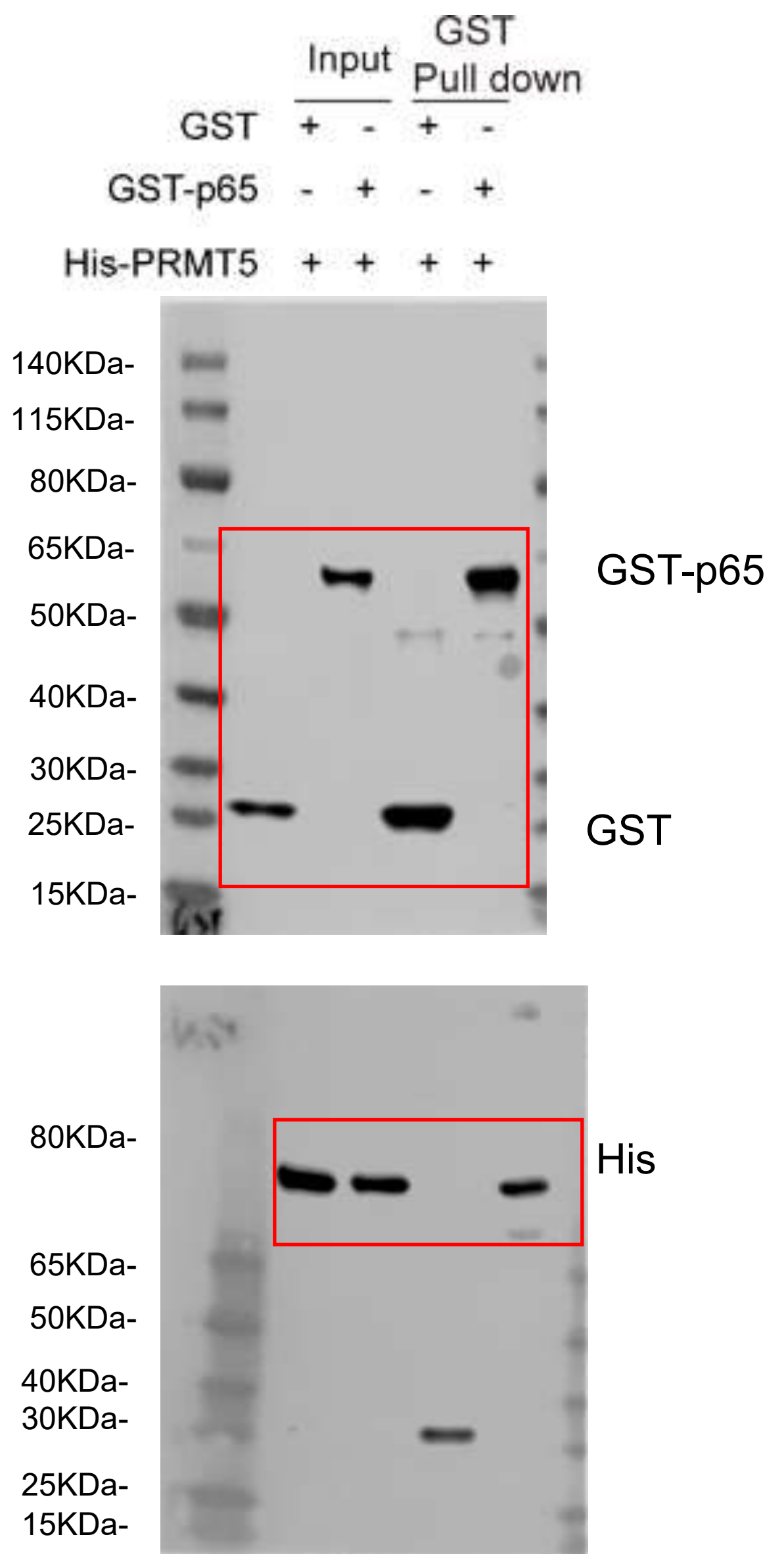

Note:Marker above 65kDa has a little bit degradation

Thermo Scientific PageRuler Protein Marker #26616 in 4-12% Bis-Tris Gel

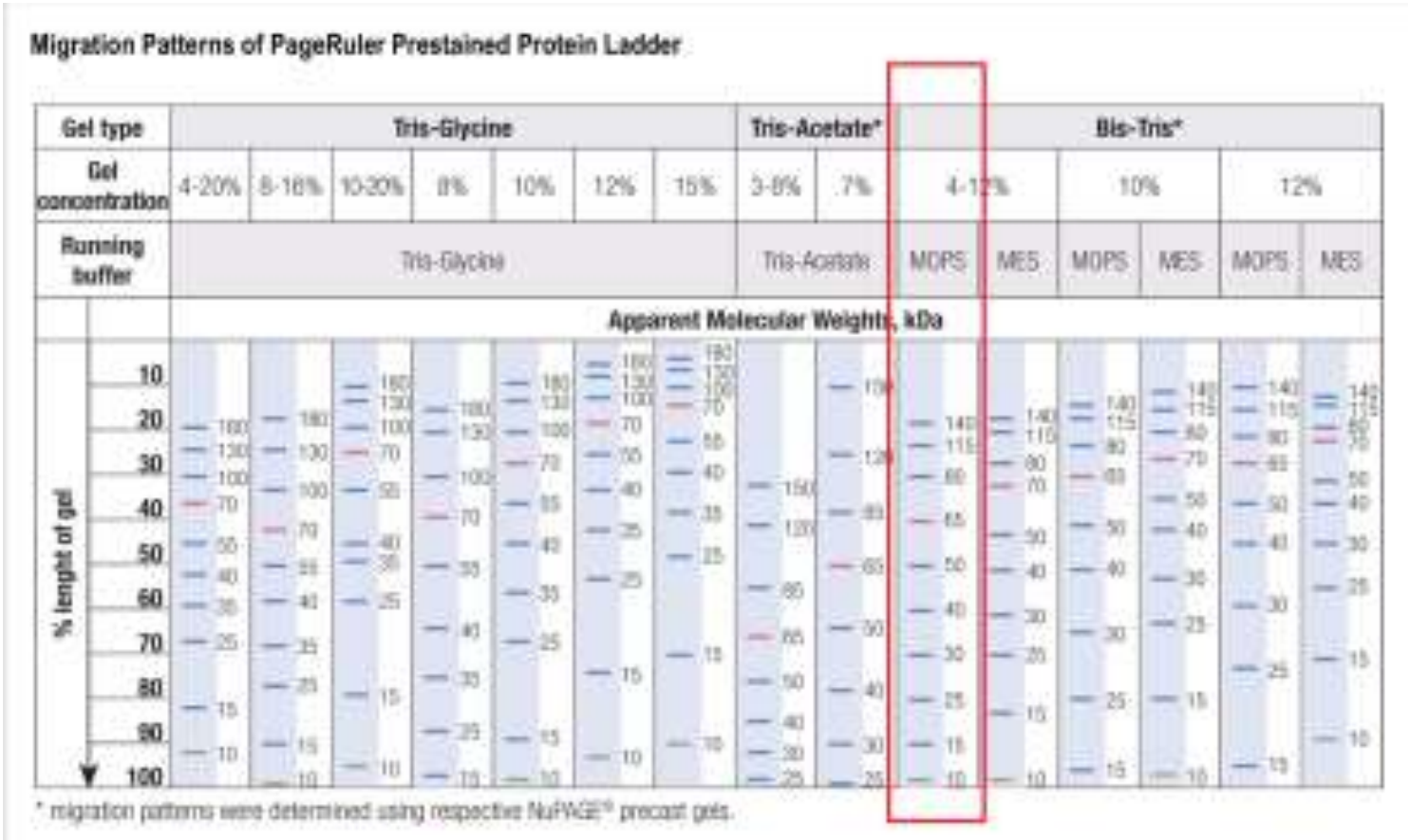

Fig4G and FigS4A

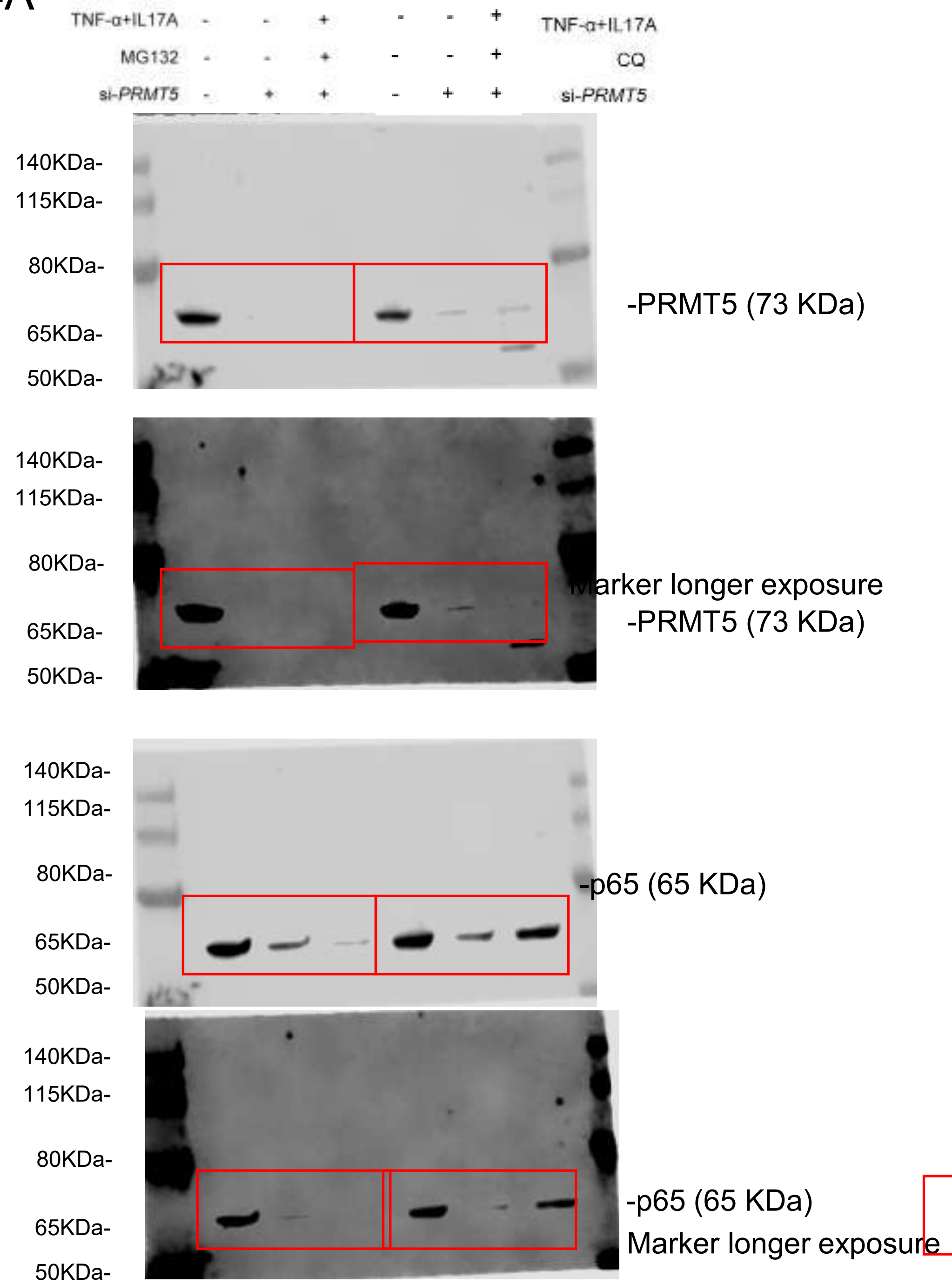

Thermo Scientific PageRuler Protein Marker
#26616 in 4-12% Bis-Tris Gel

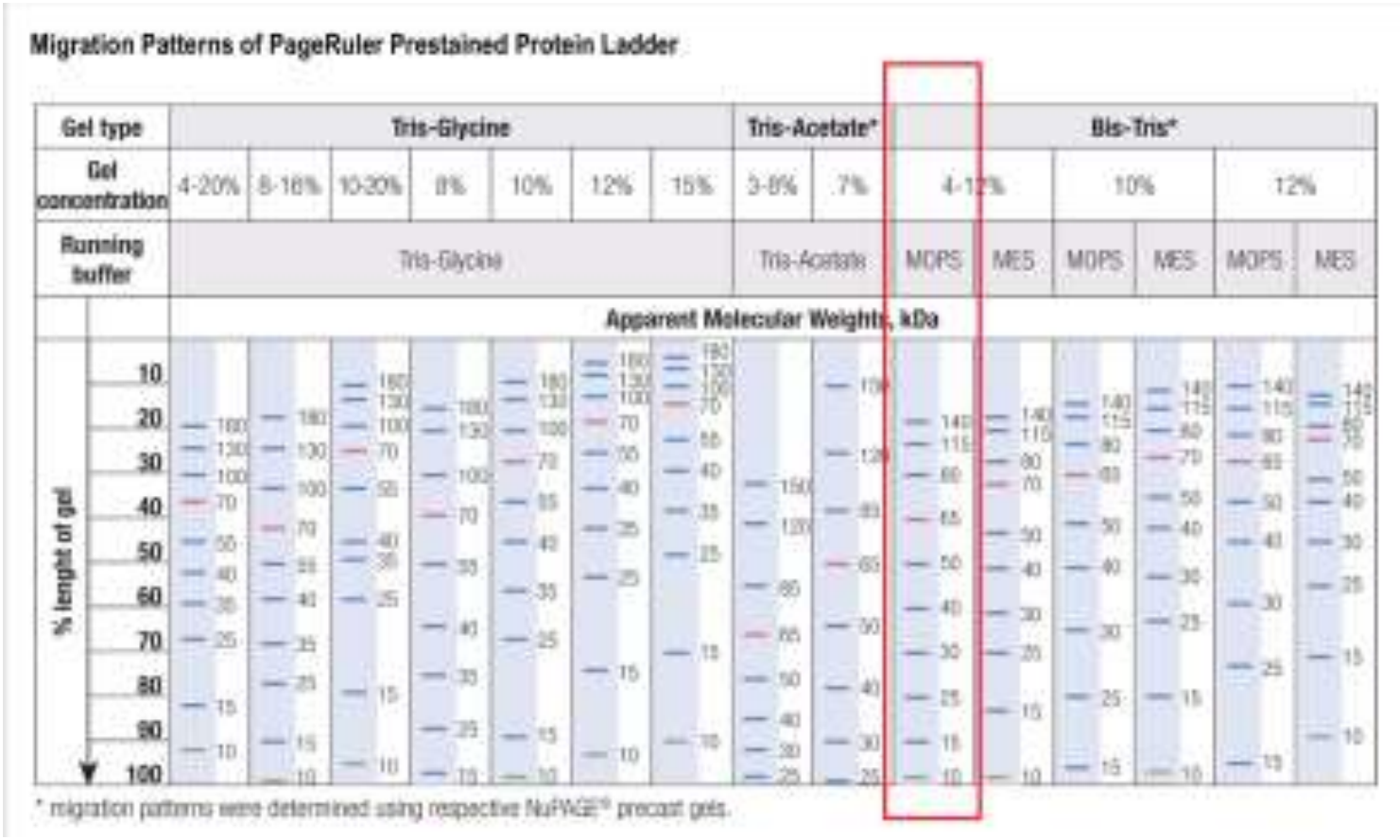

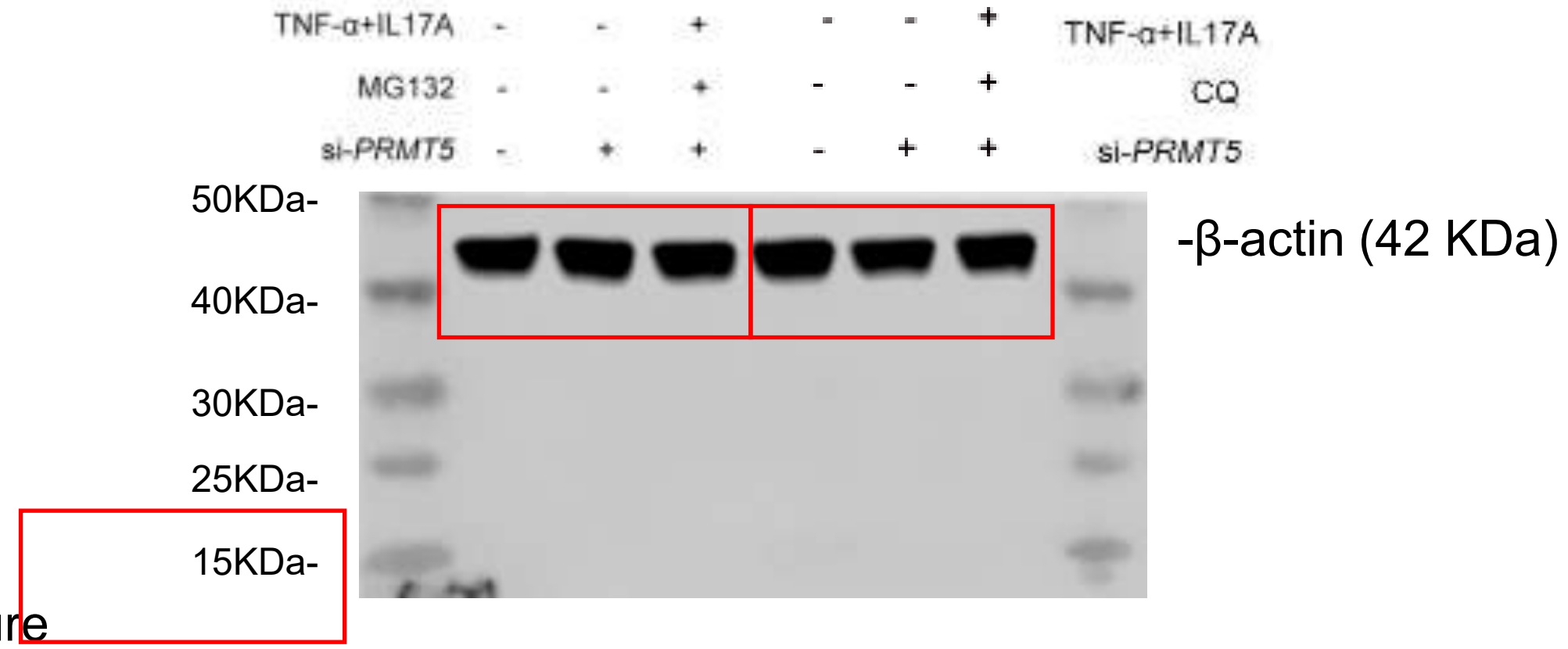

Fig5D

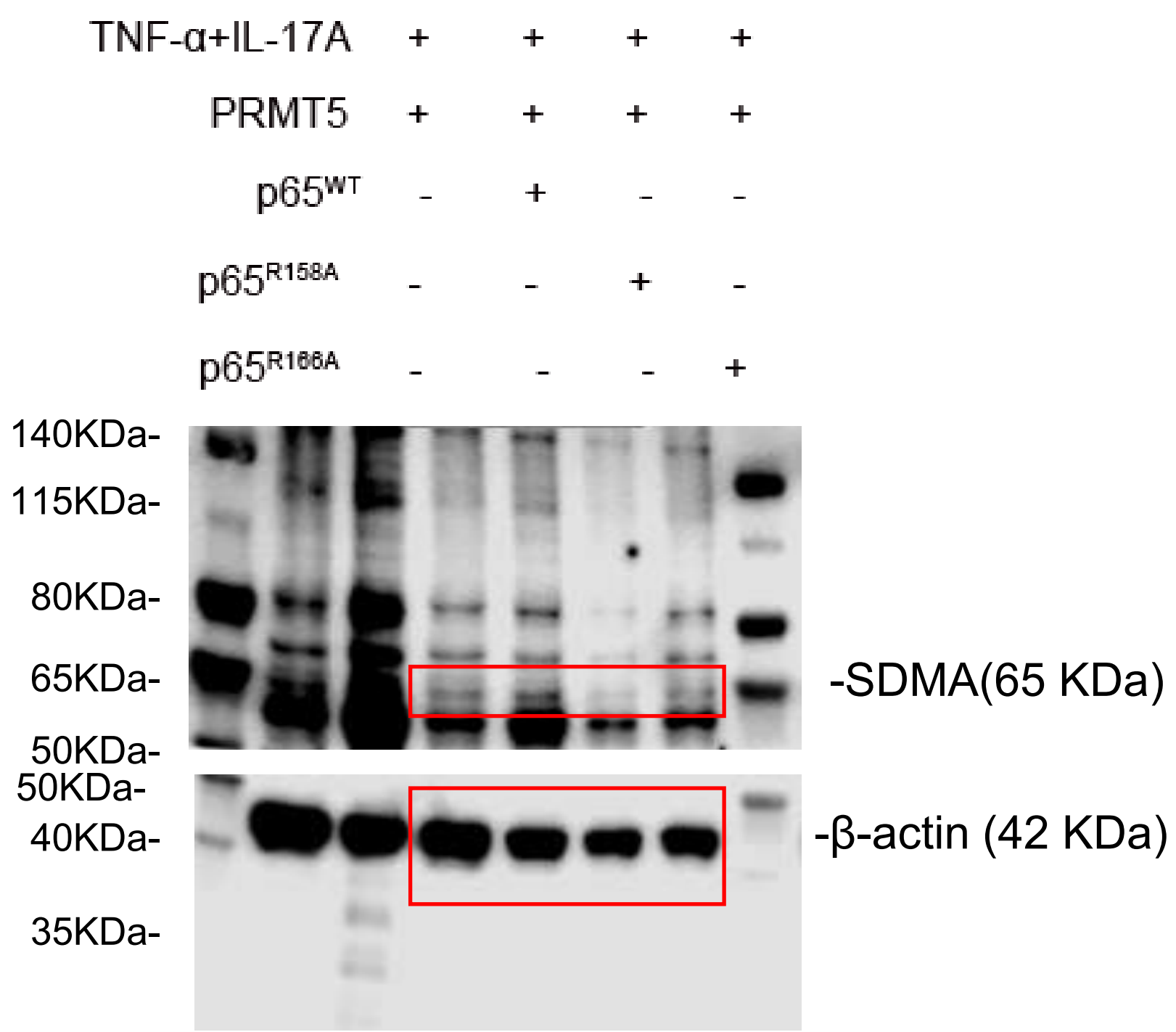

Thermo Scientific PageRuler Protein Marker
#26616 in 4-12% Bis-Tris Gel

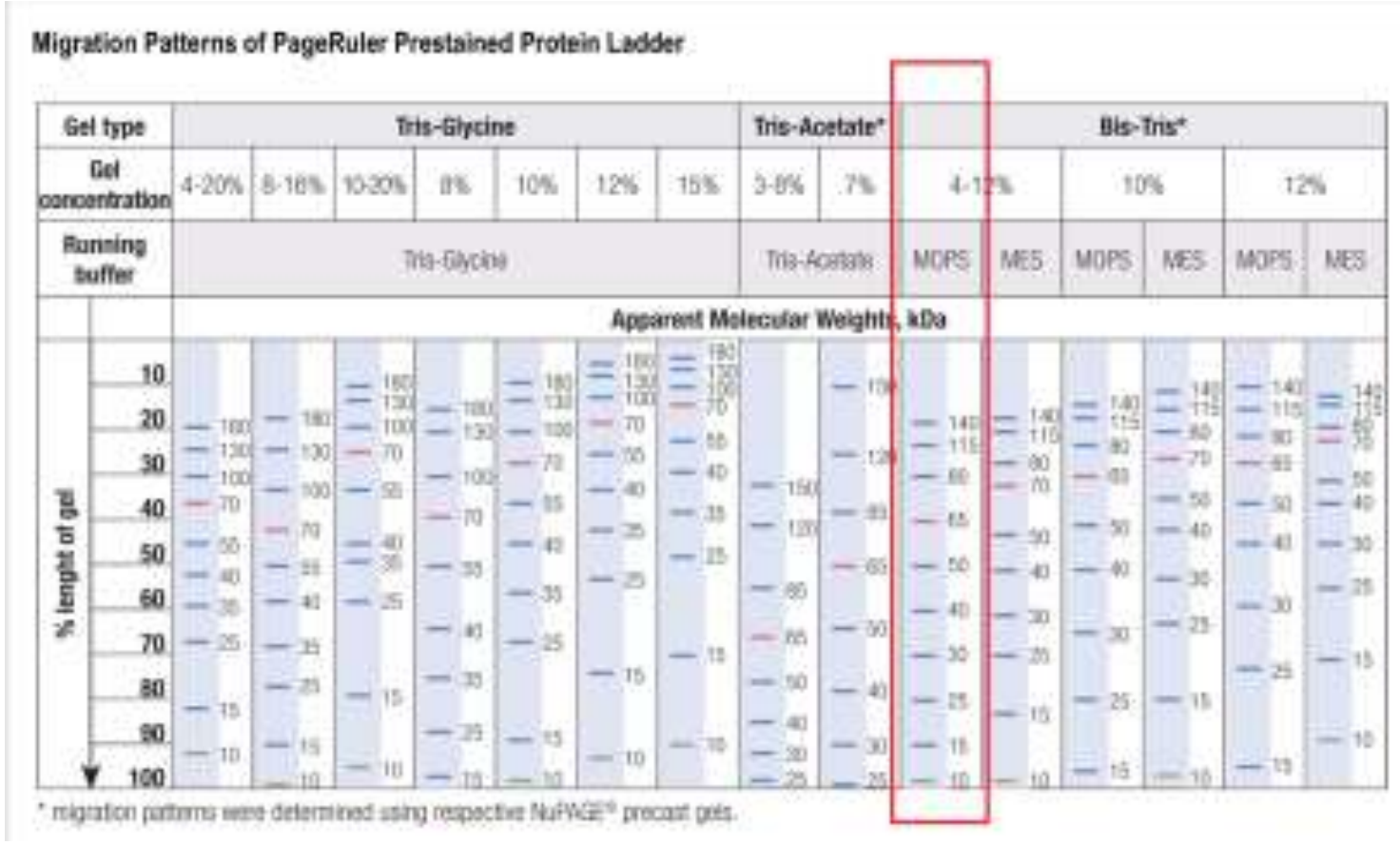

FigS2A

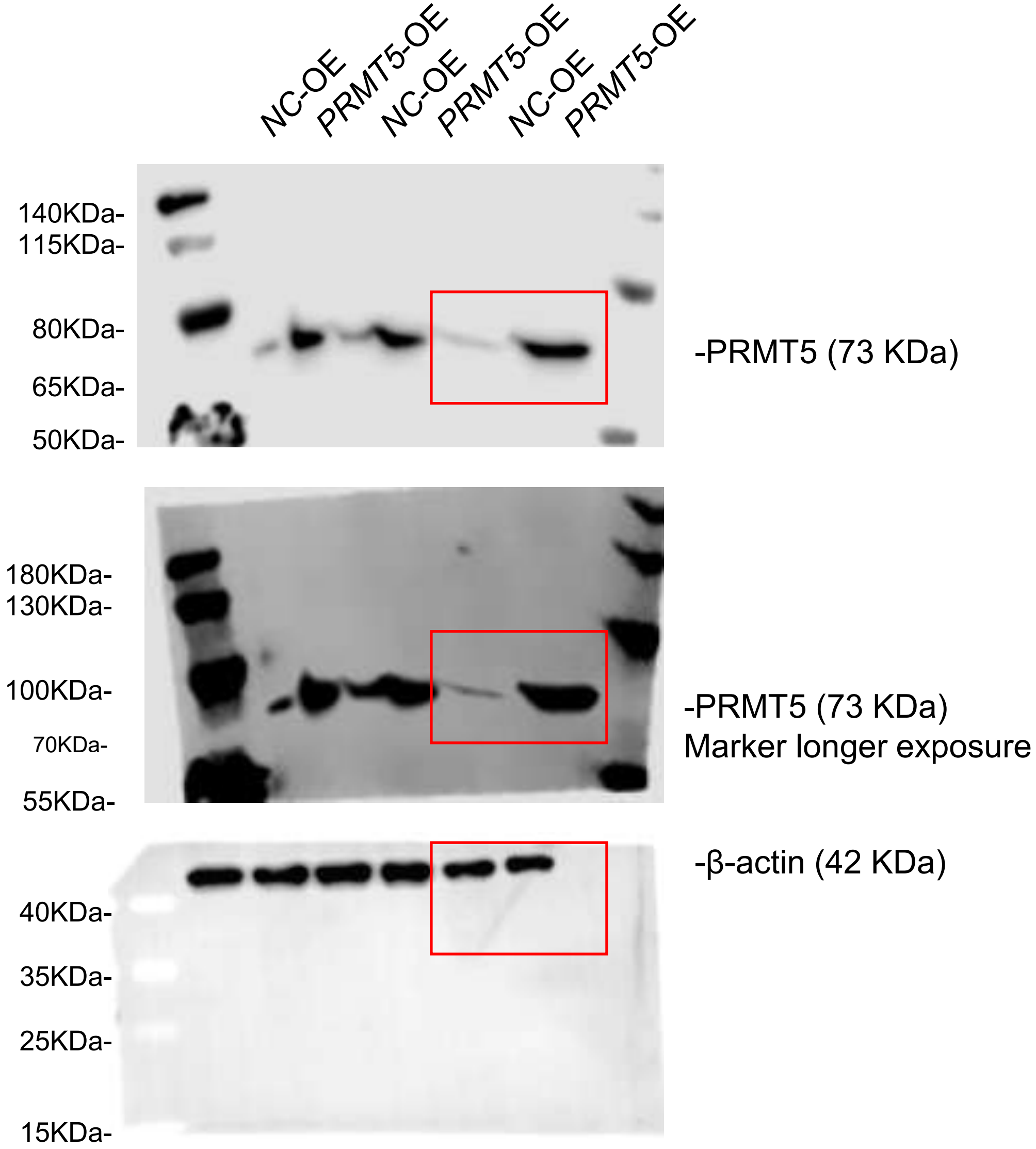

PRMT5
Thermo Scientific PageRuler Protein Marker
#26616 in 4-12% Bis-Tris Gel

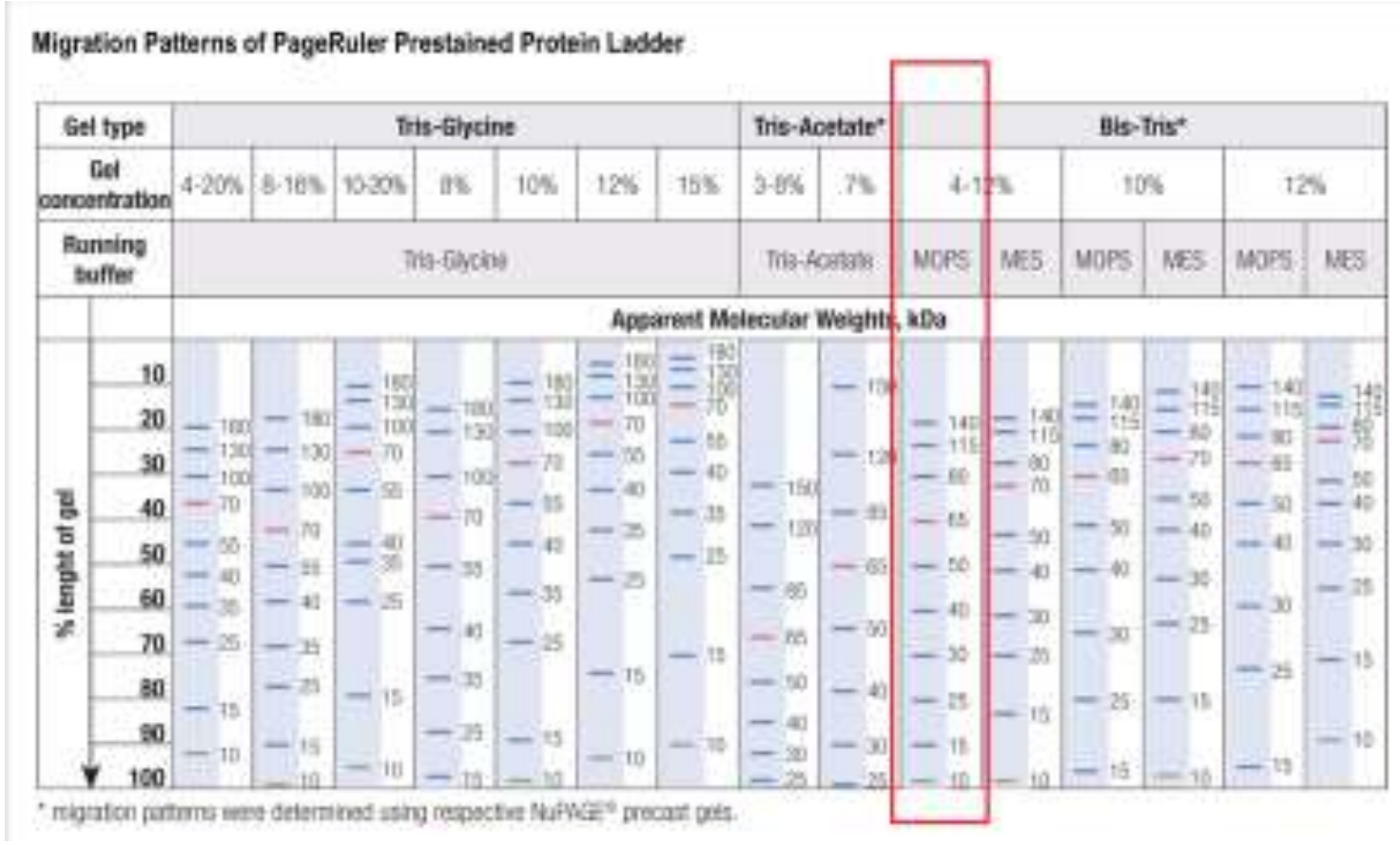

β-actin
Thermo Scientific PageRuler Protein Marker
#26616 in 10% Tris-Glycine Gel

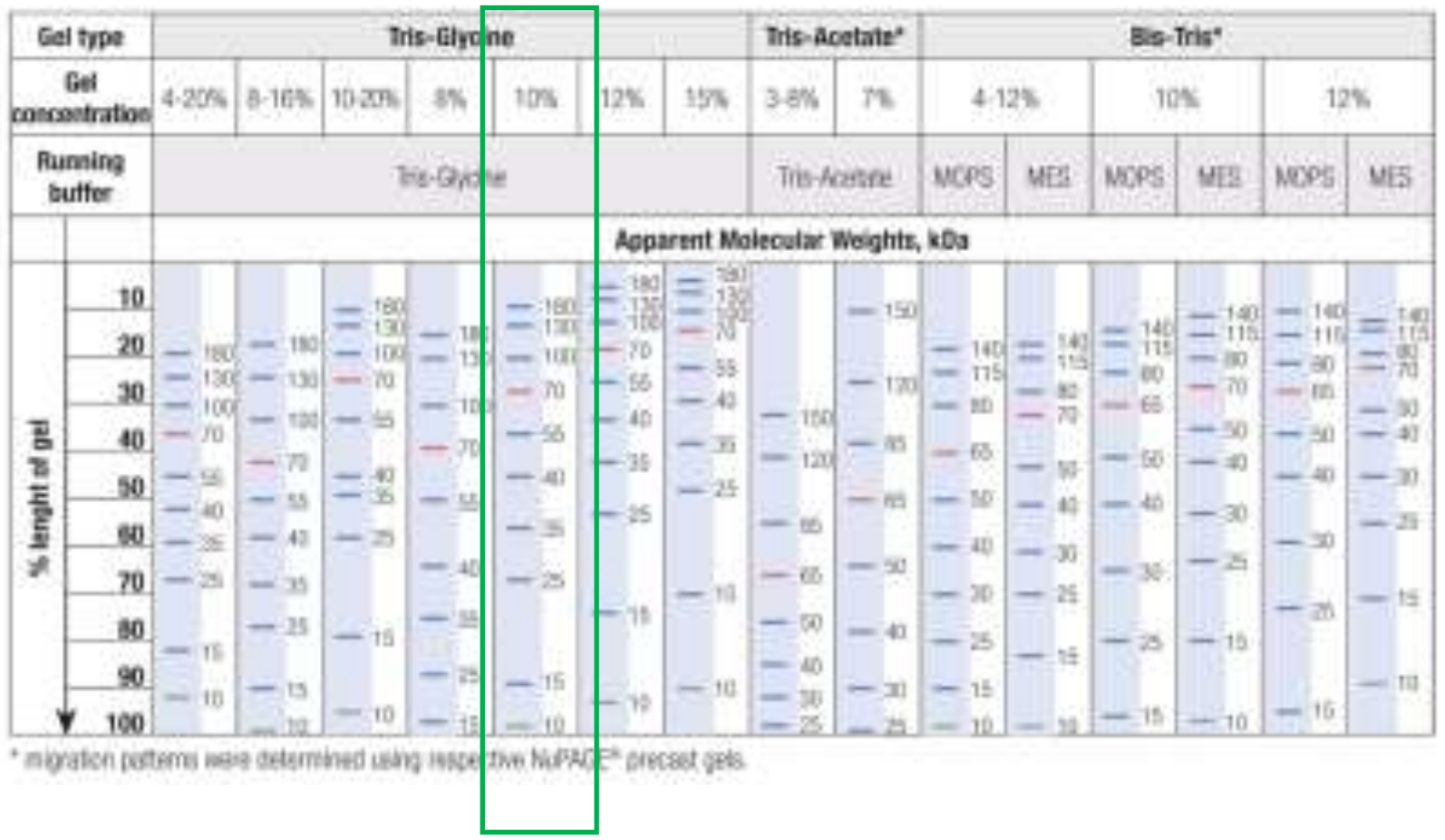

FigS3A

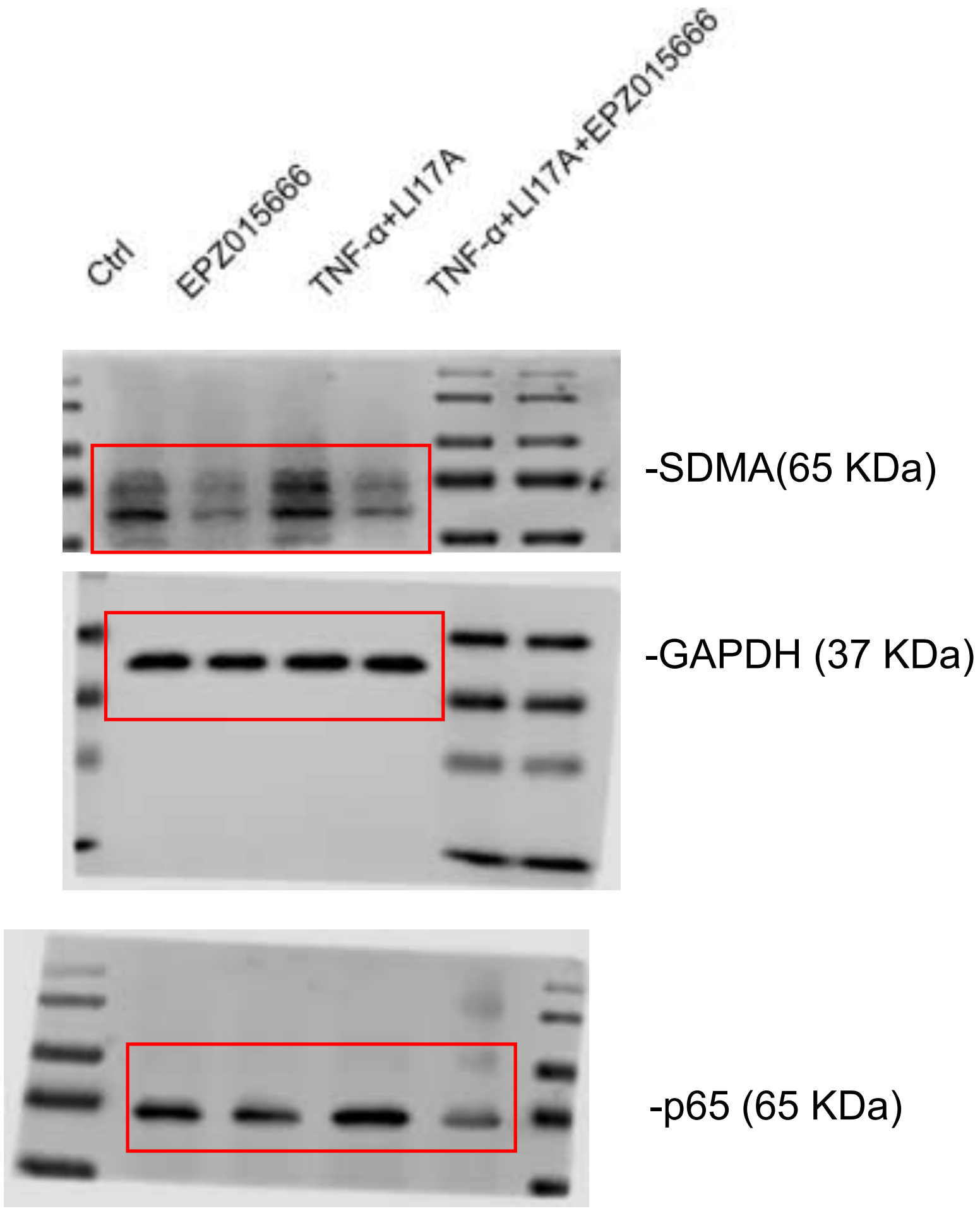

Thermo Scientific PageRuler Protein Marker
#26616 in 4-12% Bis-Tris Gel

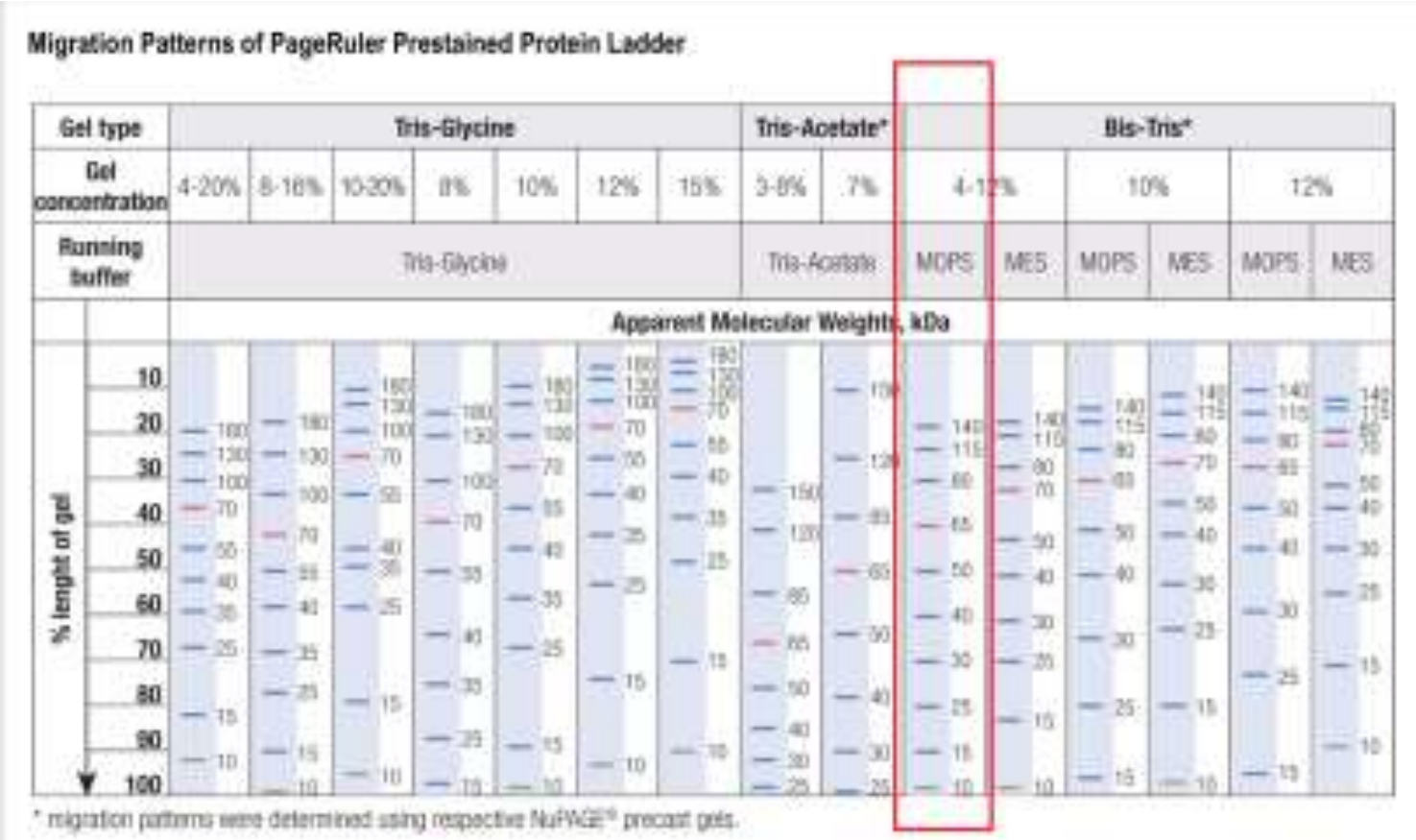

FigS3B

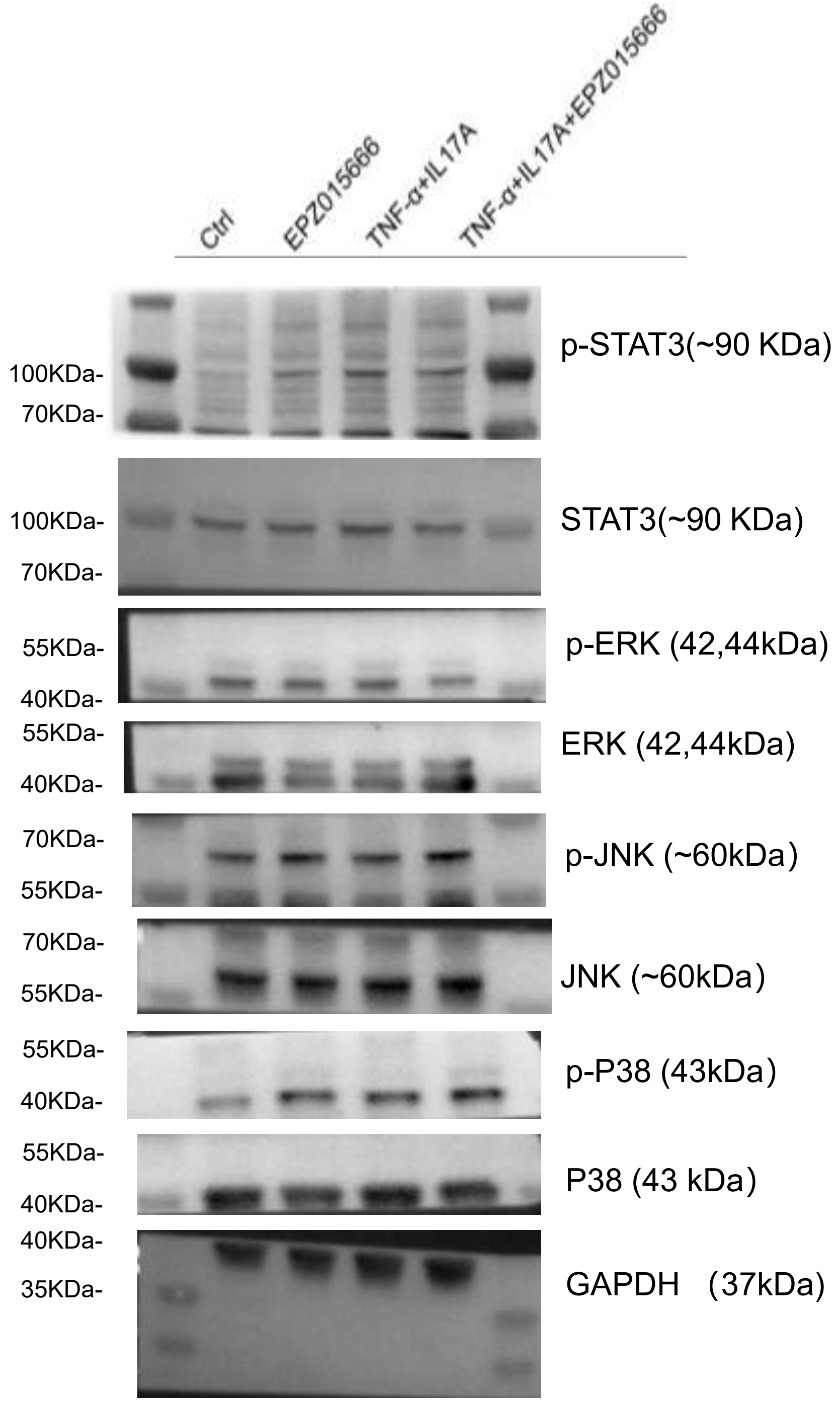

Thermo Scientific PageRuler Protein Marker
#26616 in 10% Tris-Glycine Gel

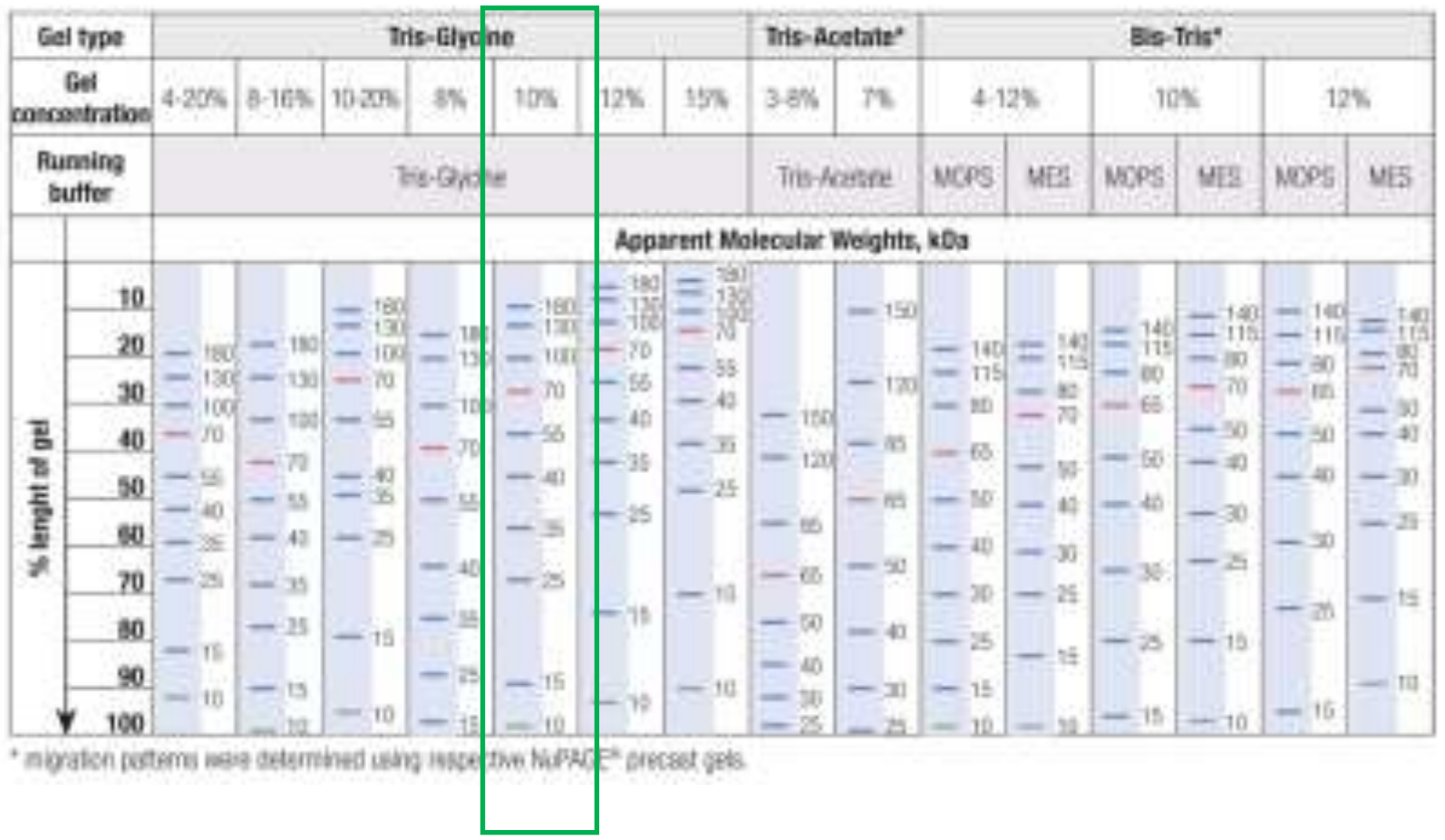

FigS5B

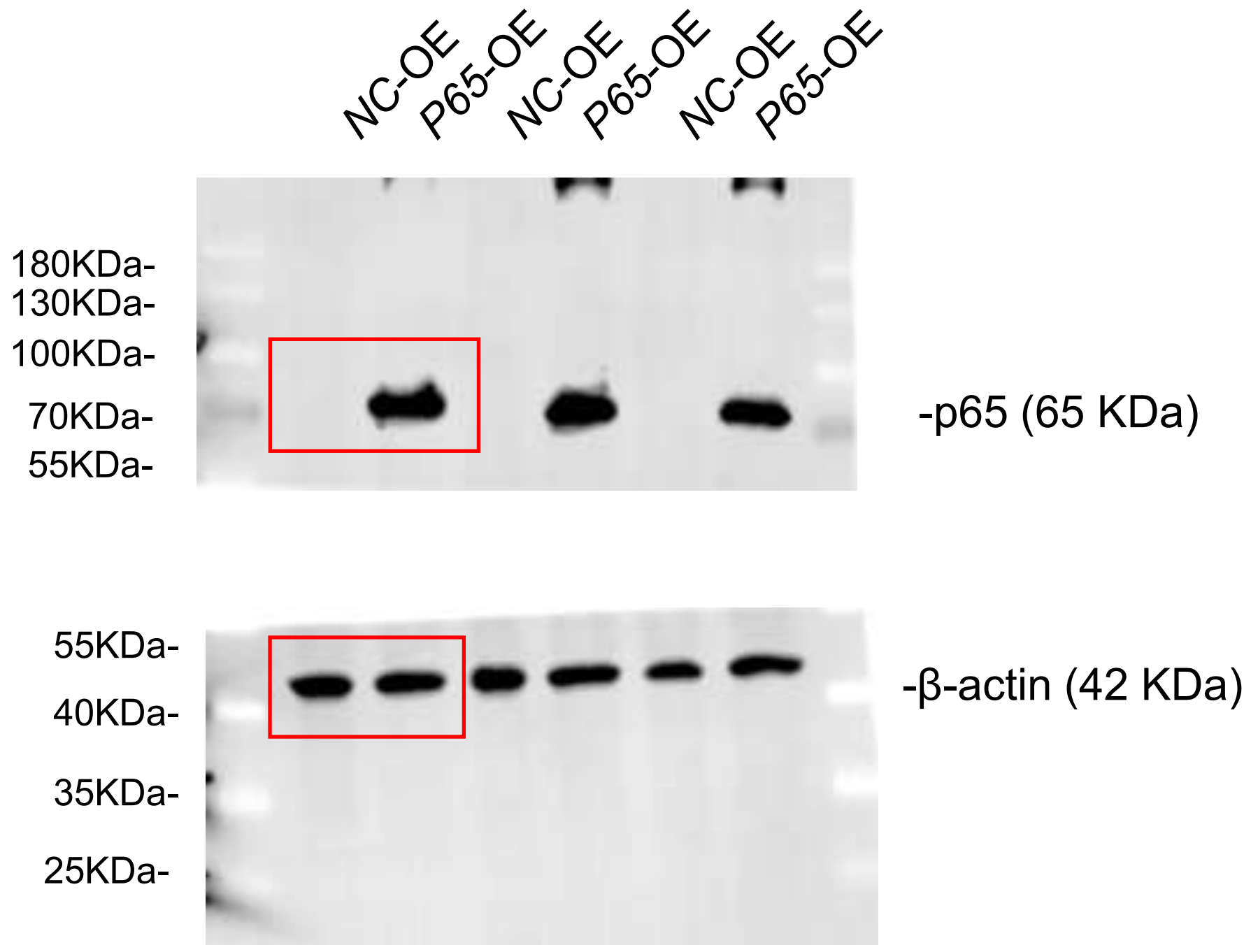

Thermo Scientific PageRuler Protein Marker
#26616 in 10% Tris-Glycine Gel

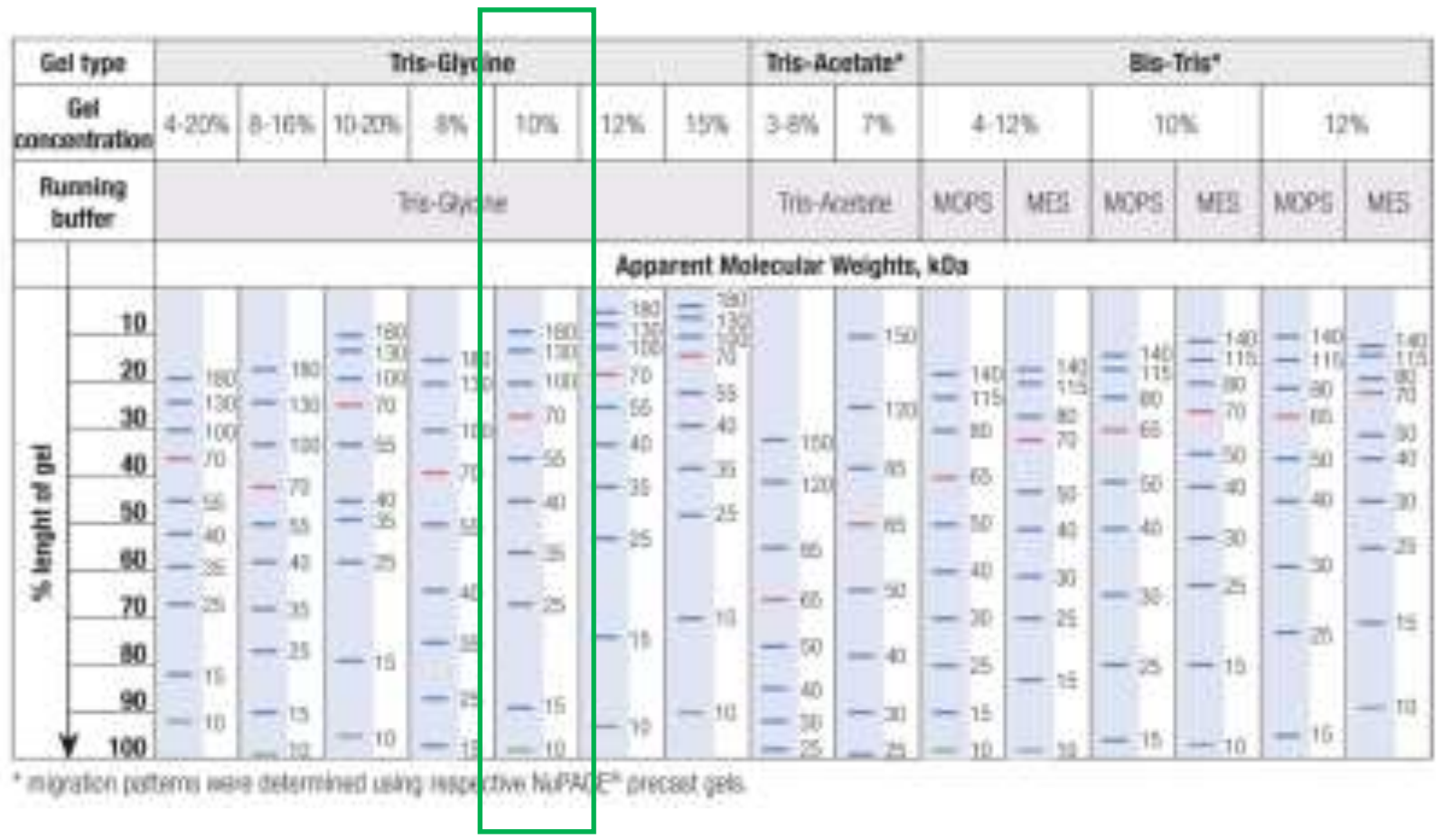

FigS5D

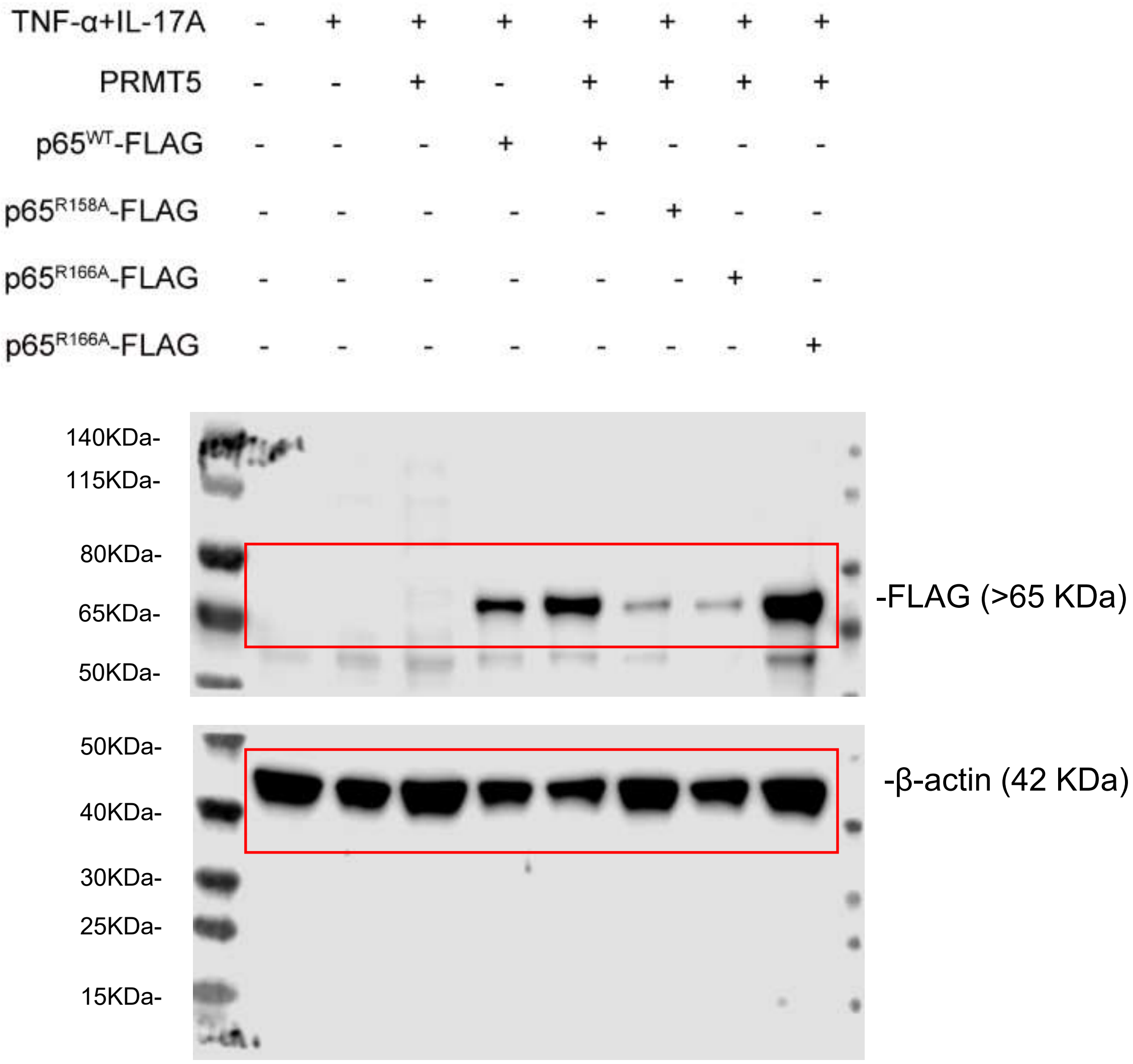

Thermo Scientific PageRuler Protein Marker
#26616 in 4-12% Bis-Tris Gel

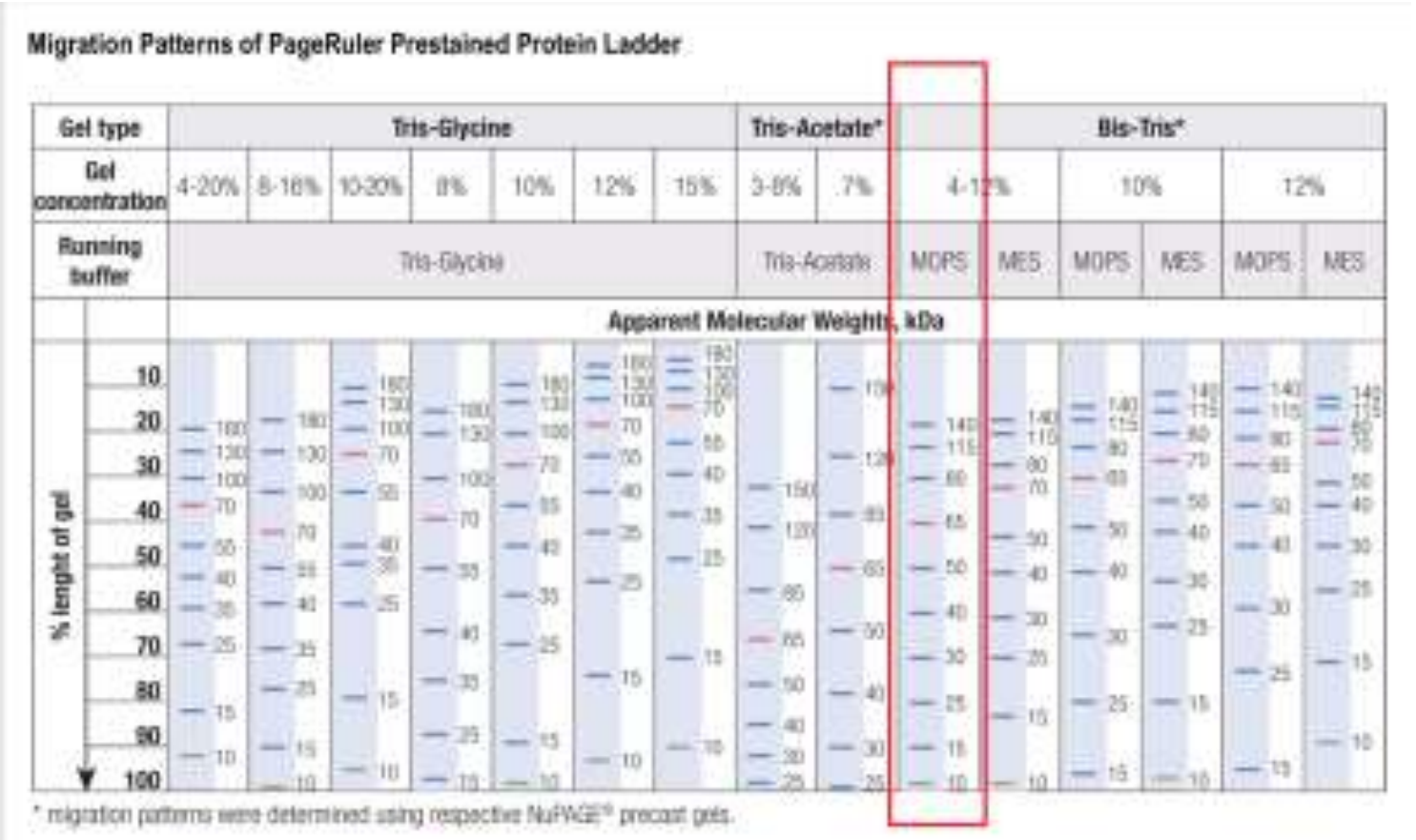

Supplement: Supplementary file 1 [file 10020_2026_1558_MOESM1_ESM.pdf]
