# Supplementary material for: PRMT5 promotes psoriatic inflammation through mediating dimethylation of p65 in keratinocytes
Source: Mol Med. 2026 Jul 10;32:172. doi: 10.1186/s10020-026-01558-6 (PMC13637110; doi:10.1186/s10020-026-01558-6)
Supplement: Supplementary file 2 [file 10020_2026_1558_MOESM2_ESM.docx]

**FigureS1. Single-cell expression profiling of PRMT5 in psoriatic skin.**

A. Dot plot showing PRMT5 expression across major skin cell types. Dot size indicates the percentage of expressing cells; color intensity reflects scaled average expression.

B. Violin plots showing PRMT5 expression in healthy control (HC), peritumoral normal (PN), and psoriatic lesion (PL) samples across nine cell types.

Data are presented as mean ± SEM. Statistical significance was determined by Wilcoxon rank-sum test (B).ns, not statistically significant. **p* < 0.05, ***p* < 0.01, ****p* < 0.001, *****p* < 0.0001.

**Figure S2. PRMT5 overexpression partially rescues EPZ015666-suppressed inflammatory gene expression in TNF-α+IL-17A-stimulated keratinocytes.**

A. Western blot analysis confirming PRMT5 overexpression (PRMT5-OE) in keratinocytes compared to negative control (NC-OE), with quantification normalized to β-actin (n = 6).

B. Relative mRNA expression of IL23A, CXCL1, and CXCL3 in keratinocytes across four groups: NC, TNF-α+IL-17A, TNF-α+IL-17A+EPZ015666, and TNF-α+IL-17A+EPZ015666+PRMT5-OE.

Data are presented as mean ± SEM (N = 4 for A; N=3 for B). Statistical significance was determined by one-way ANOVA followed by Tukey's multiple comparisons test. ns, not statistically significant. **p* < 0.05, ***p* < 0.01, ****p* < 0.001, *****p* < 0.0001.

**Figure S3. EPZ015666 suppresses PRMT5-mediated p65 methylation without affecting STAT3 and MAPK signaling pathways in keratinocytes.**

A. Representative immunoblot of p65 and SDMA protein levels in keratinocytes treated with EPZ015666, TNF-α+IL-17A, or TNF-α+IL-17A+EPZ015666 (left), with quantification normalized to GAPDH (right).

B. Representative immunoblot of phosphorylated and total protein levels of STAT3, ERK, JNK, and P38 under the same treatment conditions(left), with quantification of phosphorylation ratios (right).

Data are presented as mean ± SEM (N=3 for A-B). Statistical significance was determined by one-way ANOVA followed by Tukey's multiple comparisons test. ns, not statistically significant. **p* < 0.05, ***p* < 0.01, ****p* < 0.001, *****p* < 0.0001.

**Figure S4. Proteasomal inhibition does not rescue PRMT5 knockdown-induced p65 degradation in keratinocytes.**

A.Representative immunoblot of p65 and PRMT5 protein levels in keratinocytes treated with si-*PRMT5* in the presence or absence of the proteasome inhibitor MG132 under TNF-α+IL-17A stimulation (left), with quantification normalized to β-actin (right).

Data are presented as mean ± SEM (N=3 for A). Statistical significance was determined by one-way ANOVA followed by Tukey's multiple comparisons test. ns, not statistically significant. **p* < 0.05, ***p* < 0.01, ****p* < 0.001, *****p* < 0.0001.

**Figure S5 PRMT5 Catalyzes p65 Methylation at R336, which does not contribute to its pro-inflammatory function.**

A. Representative LC-MS/MS spectra identifying the symmetric dimethylation sites of p65 at arginine residues R336.

B. Reprensentative immunoblot of p65 (upper) and quantification (lower) in human primary keratinocytes transfected with *RELA* OE or *NC* OE.

C. qRT-PCR analysis of inflammation-related genes expression in primary human keratinocytes co-transfected with PRMT5 and either *p65*^WT^ or *p65^336A^*.

D. Representative immunoblot of FLAG-tagged p65 protein levels in keratinocytes co-transfected with *PRMT5* and *p65^WT-^FLAG, p65^R158A^-FLAG, p65^R166A^-FLAG, or p65^R336A^-FLAG* under TNF-α+IL-17A stimulation (left) with quantification normalized to β-actin (right).

Data are presented as mean ± SEM (N=3 for B-D). Statistical significance was determined by two-tailed unpaired Student’s t test (B), one-way ANOVA followed by Tukey's multiple comparisons test (C-D). ns, not statistically significant. **p* < 0.05, ***p* < 0.01, ****p* < 0.001, *****p* < 0.0001.

**Figure S6 PRMT5 inhibition attenuates psoriasis-like skin lesions in the IL-23-induced murine psoriasis model.**

A. Representative dorsal skin photographs of mice in the Ctrl, IL-23, and IL-23+EPZ015666 groups.

B. Representative H&E staining images of dorsal skin sections. Scale bar, 100 μm.

C. Quantification of epidermal thickness across the three groups.

Data are presented as mean ± SEM (N=5-7 for C). Statistical significance was determined by one-way ANOVA followed by Tukey's multiple comparisons test. ns, not statistically significant. **p* < 0.05, ***p* < 0.01, ****p* < 0.001, *****p* < 0.0001.
